# Larger Than Life: Isolation and Genomic Characterization of a Jumbo Phage That Infects the Bacterial Plant Pathogen, *Agrobacterium tumefaciens*

**DOI:** 10.3389/fmicb.2018.01861

**Published:** 2018-08-14

**Authors:** Hedieh Attai, Maarten Boon, Kenya Phillips, Jean-Paul Noben, Rob Lavigne, Pamela J. B. Brown

**Affiliations:** ^1^Division of Biological Sciences, University of Missouri, Columbia, MO, United States; ^2^Laboratory of Gene Technology, KU Leuven, Leuven, Belgium; ^3^Biomedical Research Institute and Transnational University Limburg, Hasselt University, Hasselt, Belgium

**Keywords:** *Agrobacterium*, jumbo bacteriophage, mass spectrometry, genomics, biocontrol, Atu_ph07

## Abstract

*Agrobacterium tumefaciens* is a plant pathogen that causes crown gall disease, leading to the damage of agriculturally-important crops. As part of an effort to discover new phages that can potentially be used as biocontrol agents to prevent crown gall disease, we isolated and characterized phage Atu_ph07 from Sawyer Creek in Springfield, MO, using the virulent *Agrobacterium tumefaciens* strain C58 as a host. After surveying its host range, we found that Atu_ph07 exclusively infects *Agrobacterium tumefaciens*. Time-lapse microscopy of *A. tumefaciens* cells subjected to infection at a multiplicity of infection (MOI) of 10 with Atu_ph07 reveals that lysis occurs within 3 h. Transmission electron microscopy (TEM) of virions shows that Atu_ph07 has a typical *Myoviridae* morphology with an icosahedral head, long tail, and tail fibers. The sequenced genome of Atu_ph07 is 490 kbp, defining it as a jumbo phage. The Atu_ph07 genome contains 714 open reading frames (ORFs), including 390 ORFs with no discernable homologs in other lineages (ORFans), 214 predicted conserved hypothetical proteins with no assigned function, and 110 predicted proteins with a functional annotation based on similarity to conserved proteins. The proteins with predicted functional annotations share sequence similarity with proteins from bacteriophages and bacteria. The functionally annotated genes are predicted to encode DNA replication proteins, structural proteins, lysis proteins, proteins involved in nucleotide metabolism, and tRNAs. Characterization of the gene products reveals that Atu_ph07 encodes homologs of 16 T4 core proteins and is closely related to Rak2-like phages. Using ESI-MS/MS, the majority of predicted structural proteins could be experimentally confirmed and 112 additional virion-associated proteins were identified. The genomic characterization of Atu_ph07 suggests that this phage is lytic and the dynamics of Atu_ph07 interaction with its host indicate that this phage may be suitable for inclusion in a phage cocktail to be used as a biocontrol agent.

## Introduction

Bacteriophages, or phages, are the most abundant biological entities on the planet (Clokie et al., 2011). Phages are viruses that specifically infect bacteria, often causing lysis. Phage-mediated host cell lysis of bacteria impacts environments both directly through release of dissolved organic carbon and micronutrients and indirectly by modulation of the microbial communities (Srinivasiah et al., 2008). Phages also contribute to horizontal gene transfer and host cell evolution. Bacteria-phage coevolution significantly drives gene diversity and evolution (Koskella and Brockhurst, 2014), providing both bacteria and phages with genes necessary to thrive in their environments. The diversity and vast number of phage genes coupled with limited functional characterization results in the presence of many predicted proteins of unknown function (Hatfull, 2015). Since characterization of phages and their proteins will provide biological insights, some of which can be leveraged to address current challenges in medicine and agriculture, research on phage biology, phage-host interactions, and phage-derived enzymes has recently reemerged (Santos et al., 2018).

Phage cocktails have successfully been deployed as a form of biocontrol against plant pathogens, including *Xanthomonas* species, *Ralstonia solanacearum, Pseudomonas syringae*, and *Dickeya solani* (Adriaenssens et al., 2012; Rombouts et al., 2016; Buttimer et al., 2017b). *Agrobacterium tumefaciens* is a Gram-negative bacterium that causes crown gall disease in flowering plants (Escobar and Dandekar, 2003) and phage cocktails may be a viable option to improve biocontrol of this phytopathogen; however, there are only three well-characterized *Agrobacterium* phages: Atu_ph02, Atu_ph03, and 7-7-1 (Kropinski et al., 2012; Attai et al., 2017). When searching for additional lytic phages with potential to serve as biocontrol agents against *A. tumefaciens*, we isolated a unique jumbo phage, Atu_ph07, with a dsDNA genome size of 490,380 bp.

Jumbo phages have genomes exceeding 200 kbp (Hendrix, 2009) and are less frequently isolated since they are often eliminated during common size-exclusion isolation methods due to their large size (Yuan and Gao, 2017). Most jumbo phages are members of the *Myoviridae* family and contain visible tails. The largest known phage genome belongs to *Bacillus* phage G at 497 kbp (Donelli et al., 1975), followed by *Salicola* phage SCTP-2 at 440 kbp, *Xanthomonas* phage XacN1 at 384 kbp (Yoshikawa et al., 2018), *Pectobacterium* phage CBB at 378 kbp (Buttimer et al., 2017a), *Cronobacter* phage vB_CsaM_GAP32 at 358 kbp (Abbasifar et al., 2014), and *Serratia* phage BF at 357 kbp (Casey et al., 2017). Recently, some of these T4-like jumbo phages (CBB, vB_CsaM_GAP32, BF, vB_KleM-Rak2, K64-1, 121Q, vB_Eco_slurp01, PBECO4) were classified into a new phylogenetic clade called “Rak2-like viruses” (Buttimer et al., 2017a; Yoshikawa et al., 2018), named after Enterobacteria phage Rak2 (Simoliunas et al., 2013).

In this work, we use phenotypic, genomic, and proteomic approaches to characterize phage Atu_ph07 (formal name according to Kropinski et al., 2009: vB_AtuM_Atu_ph07). Based upon comparative genome analysis and phylogenetic analysis, Atu_ph07 clusters just outside the Rak2-like phages. Though most Atu_ph07 ORFs encode as-yet uncharacterized hypothetical proteins, this work identifies functions for some key proteins, including experimentally validated structural proteins, and compares those proteins to homologs in related phages.

## Materials and methods

### Bacterial strains and culture conditions

Strains used in this study are shown in Table 1. *Agrobacterium tumefaciens* strains were cultured in Lysogeny Broth (LB), with the exception of *A. tumefaciens* strain LBA4404, which was grown in yeast mannitol (YM) medium. *Agrobacterium vitis* was cultured using potato dextrose media (Difco), *Rhizobium rhizogenes* was grown in mannitol glutamate yeast (MGY) medium and *Caulobacter crescentus* was grown in peptone-yeast extract (PYE) medium (Poindexter, 1964). *Sinorhizobium meliloti* was grown in LB (Weidner et al., 2013). These strains were grown at 28°C. *Escherichia coli* was grown in LB at 37°C. Liquid cultures were grown with shaking and solid medium was prepared with 1.5% agar.

**Table 1:** Bacterial strains used in this study.

**Strain or plasmid**	**Relevant characteristics**	**Growth medium**	**Reference or source**
***A. tumefaciens*** **STRAINS**			
C58	Nopaline type strain; pTiC58; pAtC58	LB	Watson et al., 1975
EHA105	C58 derived, succinamopine strain, T-DNA deletion derivative of pTiBo542	LB	MU plant transformation core facility
GV3101	C58 derived, nopaline strain	LB	MU plant transformation core facility
NTL4	C58 derived, nopaline-agrocinopine strain, Δ*tetRA*	LB	Luo et al., 2001
AGL-1	C58 derived, succinamopine strain, T-DNA deletion derivative of pTiBo542 ΔrecA	LB	MU plant transformation core facility
LBA4404	Ach5 derived, octopine strain, T-DNA deletion derivative of pTiAch5	YM	MU plant transformation core facility
Chry5	Succinamopine strain, pTiChry5	LB	Bush and Pueppke, 1991
LMG215	*Agrobacterium* biovar 1, genomospecies 4, isolated from hops in 1928	LB	Chang lab at Oregon State University
LMG232	*Agrobacterium* biovar 1, genomospecies 1, isolated from beet in 1963	LB	Chang lab at Oregon State University
A74a	*Agrobacterium* biovar 1, genomospecies 8, isolated from Pennsylvania lavender in 2003	LB	Chang lab at Oregon State University
06-777-2L	*Agrobacterium* biovar 1, genomospecies 7, isolated from Marguerite Daisy in 2006	LB	Chang lab at Oregon State University
**OTHER BACTERIAL STRAINS**			
*A. vitis* S4	Vitopine strain, pTiS4, pSymA, pSymB	Potato dextrose	Slater et al., 2009
*Rhizobium rhizogenes* D108/85	*Agrobacterium* biovar 2, isolated from Michigan blueberry 1985	MGY	Chang lab at Oregon State University
*Caulobacter crescentus* CB15	Alphaproteobacterium	PYE	Nierman et al., 2001
*Sinorhizobium meliloti* 1021	Rhizopine strain, pSymA, pSymB, pRme41a	LB	Weidner et al., 2013
*Escherichia coli* DH5α	Gammaproteobacterium	LB	Life Technologies

### Clonal isolation of bacteriophage Atu_ph07

Atu_ph07 was isolated from Sawyer Creek in Springfield, MO using *Agrobacterium tumefaciens* strain C58 as the host. Atu_ph07 was isolated using an enrichment protocol (Santamaría et al., 2014) adapted as described previously (Attai et al., 2017).

### Partial purification of virions

Virions were concentrated and partially purified from 2 L lysate by polyethylene glycol (PEG) precipitation (Yamamoto et al., 1970) and differential centrifugation. All centrifugations and incubations were performed at 4°C. The starting lysate was distributed evenly into six 500-ml centrifuge bottles and centrifuged at 5,000 rpm for 20 min to pellet bacterial cells. The supernatants were poured into fresh bottles, which were centrifuged as before to pellet residual bacterial cells. The doubly-cleared supernatants were treated with DNase I (Sigma D5025) at a final concentration of 3.5 μg/ml with stirring for 1 h at room temperature to digest bacterial DNA. Solid NaCl was added to a final concentration of 0.5 M with stirring; when the salt was fully dissolved, solid PEG 8000 (Fisher BP233-1) was added gradually to a final concentration of 10% w/w with constant stirring; stirring was continued for another 2 h. The suspension was distributed evenly into six 500–ml centrifuge bottles, which were refrigerated overnight before being centrifuged at 8,000 rpm for 10 min to pellet the virions; supernatants were decanted and discarded; the bottles were centrifuged again and residual supernatants were removed by aspiration. To each bottle, 20 ml 1 × Dulbecco's phosphate-buffered saline with magnesium and calcium (DPBS; Fisher) was added and the pellets dissolved by gentle shaking at 4°C overnight.

The six dissolved pellets were distributed evenly into four 50–ml disposable plastic conical centrifuge tubes, which were centrifuged for 10 min at 5,000 rpm to pellet insoluble material. The cleared supernatants were pooled and 1/9 vol of 5 M NaCl was added. Solid PEG 8000 was added gradually with stirring to a final concentration of 10% w/w, and stirring was continued for another 1 h at room temperature. The suspension was distributed evenly into four 50–ml centrifuge tubes, which were centrifuged 20 min at 10,000 rpm to pellet virions. The supernatants were decanted and discarded, and the tubes were centrifuged again briefly. Residual supernatants were aspirated and discarded. To each tube, 15 ml DPBS was added, and the tubes were rotated overnight at 4°C to dissolve the pellets. The tubes were centrifuged for 10 min at 5,000 rpm to pellet insoluble material. The supernatants combined and 0.6 ml of sterile 10–mM phenol red (pH ~7) was added to color the solution cherry-red. This solution was layered onto 2–ml cushions of 5% w/w sucrose in DPBS in six 14 × 89 mm Ultraclear centrifuge tubes (Beckman 331372) and centrifuged at 40,000 rpm for 20 min in a Beckman SW41Ti rotor. The clear supernatants were aspirated gently. 1 ml DPBS was added to each tube and the pellets were dissolved by periodic vortexing and standing overnight at 4°C. The dissolved pellets were transferred to six 1.5–ml microtubes, which were vortexed to complete dissolution and centrifuged at 6,000 rpm for 5 min to pellet insoluble material. The supernatants were transferred to fresh 1.5–ml microtubes, which were centrifuged at 13,000 rpm for 30 min to pellet virions. The supernatants were aspirated, the tubes were centrifuged, and the residual supernatants were aspirated. The pellets were resuspended in 1 ml DPBS by periodic vortexing and standing overnight at 4°C. Finally, the tubes were centrifuged at 6,000 rpm for 5 min to pellet insoluble material and the cleared supernatants were transferred to fresh 1.5–ml microtubes and stored at 4°C. The solutions were notably turbid. Virion concentration was estimated at 10^12^ physical particles/ml by scanning a 1/10 dilution spectrophotometrically, assuming that intact virions have about the same molar absorption coefficient at 260 nm as do naked 490,380-bp DNA molecules. The infective titer was 7 × 10^10^ plaque forming units (pfu)/ml.

### Plaque assays

Whole-plate plaque assays were performed with the soft agar overlay method (Attai et al., 2017). Briefly, 100 μl cells, grown at an optical density at 600 nm (OD_600_) of ~0.2 and diluted to OD_600_ of 0.05, were mixed with 100 μl phage for 15 min at room temperature prior to dilution to allow attachment. This mixture of cells and phage were serially diluted in LB and added to 3 ml melted 0.15 or 0.3% LB-soft agar. The solution was then overlaid onto a 1% LB-agar plate and swirled for even distribution. For host range testing, serial dilutions of phage were spotted onto a bacterial lawn. A mixture of 100 μl cells (OD_600_ of ~0.2) and 0.3% LB-soft agar was overlaid onto a 1% LB-agar plate. Once the cells solidified, 5 μl of phage dilutions were spotted onto the soft agar. Plates were incubated for 1–2 days to allow plaque formation.

### Preparation of virion DNA

Virion DNA was prepared essentially as described (Attai et al., 2017). A 500–μl portion of partially purified virions was pipetted into a 1.5–ml microfuge tube and extracted twice with neutralized phenol (liquefied phenol equilibrated twice with 1/10 vol 1 M Tris-HCl pH 8, discarding the small upper phase each time) and once with chloroform:isoamyl alcohol (24:1 v/v) as follows: 500 μl neutralized phenol or chloroform:isoamyl alcohol was added to the microtube, the microtube was vigorously vortexed, and the phases were separated by centrifugation at 13,000 rpm for 2 min; most of the lower (organic) phase was removed and discarded, the microtube was centrifuged as before, and the upper (aqueous) phase containing the DNA was transferred to a fresh microtube, taking care to avoid residual bottom layer and interphase material. Next, 40 μl 3 M sodium acetate (pH adjusted to 6 with acetic acid) and 1 ml 100% ethanol were mixed with the final extract to precipitate the DNA. The precipitate was pelleted by centrifugation at 13,000 rpm for 10 min and washed gently with 1 ml freezer-cold 70% ethanol. The pellet was air-dried, dissolved in 100 μl 1 mM Tris-HCl pH 7.5, 100 μM Na_2_EDTA, and stored at −20°C.

### Growth curves

Growth curves were performed by growing bacteria at a starting OD_600_ of 0.05 in LB. Cells were mixed with purified Atu_ph07 in liquid media at the MOIs indicated. Cell growth was measured by the culture turbidity, represented by the absorbance at OD_600_. Measurements were taken every 10 min for 36 h. Cells were grown at 28°C and shaken for 1 min prior to each reading. The OD_600_ was measured using a BioTek Synergy H1 Hybrid reader. Results were taken in quadruplicate and averaged. For host range testing, Atu_ph07 was added to cells at an MOI of 10.

### Time-lapse microscopy

*A. tumefaciens* strain C58 cells were grown to an OD_600_ of 0.2 and infected with Atu_ph07 at an MOI of 10. Infected cells were incubated at room temperature for 15 min to allow phage attachment and 1 μl infected cells were spotted on a 1% agarose pad containing LB, as previously described (Howell et al., 2017). Cells were imaged using a 60 × oil immersion objective (1.4 numerical aperture) by differential interference microscopy every 10 min for 24 h using a Nikon Eclipse TiE equipped with a QImaging Rolera EM-C^2^ 1 K electron-multiplying charge-coupled-device (EMCCD) camera and Nikon Elements imaging software.

### Transmission electron microscopy

Virion morphology was observed by applying a small volume of concentrated purified virions onto a freshly, glow-discharged carbon-coated TEM grid and negatively stained with 2% Nano-W (Nanoprobes, LLC, Brookhaven NY). Specimens were observed on a JEOL JEM-1400 transmission electron microscope at 120 kV. Capsid diameters of 100 virions were measured using ImageJ (v.2.0.0) (Schneider et al., 2012). Head lengths were measured from the top of the phage head vertex to the top of the neck (*n* = 114). Head widths were measured from the right vertex of the head to the left vertex, approximately equidistant between the top of the head vertex and top of the neck and perpendicular to the tail (*n* = 118). Tail lengths were measured from the bottom of the head vertex to the baseplate (*n* = 102). Contracted tails were also measured (*n* = 12).

### Genome sequencing and assembly

Libraries for genome sequencing were constructed from virion DNA following the manufacturer's protocol and reagents supplied in Illumina's TruSeq DNA PCR-free sample preparation kit (FC-121-3001). Briefly, 2.4 μg of DNA was sheared using standard Covaris methods to generate average fragmented sizes of 350 bp. The resulting 3′ and 5′ overhangs were converted to blunt ends by an end repair reaction using 3′-to-5′ exonuclease and polymerase activities, followed by size selection (350 bp) and purification with magnetic sample purification beads. A single adenosine nucleotide was added to the 3′ ends of the blunt fragments followed by the ligation of Illumina indexed paired-end adapters. The adaptor-ligated library was purified twice with magnetic sample purification beads. The purified library was quantified using a KAPA library quantification kit (KK4824), and library fragment sizes were confirmed by Fragment Analyzer (Advanced Analytical Technologies, Inc.). Libraries were diluted, pooled, and sequenced using a paired-end 75-base read length according to Illumina's standard sequencing protocol for the MiSeq. Library preparation and sequencing were conducted by the University of Missouri DNA Core facility.

### Genome annotation

Protein-coding regions were annotated by RAST server (Aziz et al., 2008) and PSI-BLAST (Altschul et al., 1997) with an e-value cut-off of 1e-03. Proteins of interest were analyzed by TMHMM (Krogh et al., 2001) and SignalP 4.1 (Petersen et al., 2011). The presence of tRNAs were detected by tRNAscan-SE (version 2.0) (Lowe and Chan, 2016). Codon usage was analyzed by Geneious (v.11.0.5) (Kearse et al., 2012). Pairwise (%) nucleotide identity was determined using the Mauve plugin in Geneious (Darling et al., 2004).

### 16S rRNA gene amplification

16S rRNA gene sequences were amplified by colony PCR using One*Taq* DNA Polymerase (New England Biolabs) and universal primers, 27F and 1492R (Hogg and Lehane, 1999; Turner et al., 1999). Amplified DNA was purified using the GeneJET PCR Purification Kit (Thermo Scientific) and sequenced by the MU DNA Core facility.

### Phylogenetic and gene product analysis

Homologs of the major capsid protein, large terminase subunit, and portal vertex protein were identified by BLASTp using an E-value cutoff of 1e-03. Protein alignment was performed by Geneious using ClustalW (v.2.1) and the BLOSUM matrix (Larkin et al., 2007; Kearse et al., 2012). Maximum-likelihood trees based on phylogeny (PhyML) were built using a Geneious plugin with 100 bootstrap models (Guindon et al., 2010). For the 16S rRNA tree, a ClustalW nucleotide alignment and a neighbor-joining tree were created in Geneious using the Jukes-Cantor genetic distance model. These trees were imported and annotated in iTOL (v3) (Letunic and Bork, 2016).

### SDS-PAGE and electron spray ionization mass spectrometry (ESI-MS/MS)

Starting from a PEG purified phage stock of >10^10^ pfu/ml, a protein pellet was obtained by chloroform:methanol extraction [1:1:0.75 (vol/vol/vol)]. The pellet was resuspended in loading buffer [40% Glycerol (vol/vol), 4% SDS (wt/vol), 200 mM Tris-HCl pH 6.8, 8 mM EDTA, 0.4% Bromophenol blue (wt/vol)] and heated at 95°C for 5 min before loading on a 12% SDS-PAGE gel. After separation by gel electrophoresis, virion proteins were visualized by staining in Gelcode™ Blue Safe Protein Stain (Thermo Scientific). Fragments covering the full lane of the gel were subsequently isolated and subjected to trypsin digestion as described by Shevchenko et al. (1996). The samples were then analyzed by nano-liquid chromatography-electrospray ionization tandem mass spectrometry (nanoLC-ESI-MS/MS) and peptides were identified, based on a database containing all predicted phage proteins, using the search engines SEQUEST [v 1.4.0.288] (ThermoFinnigan) and Mascot [v 2.5] (Matrix Science).

### Lysogen induction and detection assays

To test if Atu_ph07 produces lysogens, we attempted to induce C58 cells that survived Atu_ph07 infection with mitomycin C and ultra-violet (UV) irradiation. Ten survivor strains were isolated by streak-purifying 3 times on LB-agar and were confirmed to survive Atu_ph07 infection by conducting spot assays. Survivor cells were grown to OD_600_ of 0.4–0.5 and mixed with mitomycin C (Fisher) at a final concentration of 0.5 μg/ml for 2 h at 28°C with shaking or grown to an OD_600_ of 0.6–0.7 irradiated with UV for a time ranging from 3 s to 120 s. In each case, cells were centrifuged at 7,500 rpm for 10 min. The supernatant was filtered through a 0.45 μm column and centrifuged at 4,000 rpm for 10 min. The flow-through was spotted (5 μl) on a lawn of C58 (OD_600_ = 0.2, 0.3% LB-agar) and incubated overnight at 28°C to be observed for plaque formation in comparison to the Atu_ph07 control.

To find prophages in the genomes of survivor strains, we attempted to PCR amplify genes from Atu_ph07 that are not present in C58 using two sets of primers, which amplified nicotinate phosphoribosyltransferase (CDS 242) and adenine-specific methyltransferase (CDS 399). Primers to amplify nicotinate phosphoribosyltransferase (1,299 bp) were 5′ ATG ATC GAT ATC GCA ACA 3′ (forward) and 5′ TTA GAC AAT TAG AGG TGC 3′ (reverse) and adenine-specific methyltransferase (759 bp) primers were 5′ATG CAA ATT GGT AAT GGG 3′ (forward) and 5′ TTA AAA TTC AAA TAG CCC 3′ (reverse). PCR was performed using One*Taq* DNA Polymerase (New England Biolabs). DNA from Atu_ph07 or *A. tumefaciens* were used as positive and negative controls, respectively.

### Accession number

The genome sequence of Atu_ph07 has been deposited in the GenBank database with the nucleotide accession number MF403008.

## Results and discussion

### Isolation and characterization of Atu_ph07

**Figure 1:**
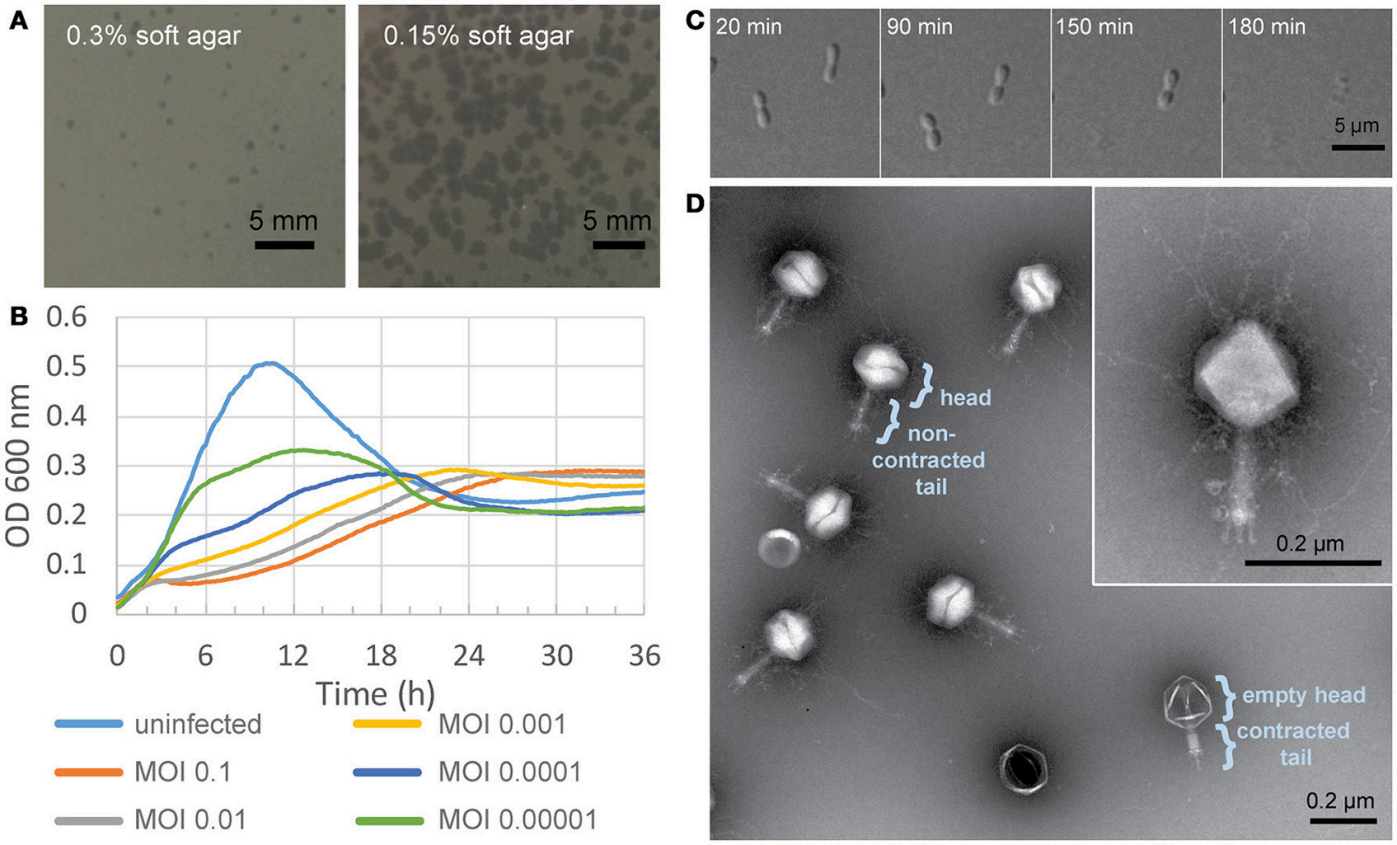
Characterization of Atu_ph07. **(A)** Atu_ph07 forms small plaques on a lawn of *A. tumefaciens* C58 on 0.3% soft agar (left) and larger plaques on 0.15% soft agar (right). **(B)** Growth curve of *A. tumefaciens* infected with Atu_ph07 at different MOIs. **(C)** Time-lapse microscopy of *A. tumefaciens* cells infected with Atu_ph07 at an MOI of 10. **(D)** TEM image of Atu_ph07 shows the phage is in the family *Myoviridae*. Inset of a single phage particle at higher magnification is shown to emphasize the presence of tail fibers and whiskers.

*Agrobacterium tumefaciens* strain C58 (Watson et al., 1975) was used as a host strain to isolate phages from environmental samples. We isolated phage Atu_ph07 from a water sample from Sawyer Creek in Springfield, MO using a modified phage enrichment protocol (Santamaría et al., 2014). Following filtration of water samples, we noticed the clearing of bacterial cultures after two rounds of incubation with C58. The presence of phage was apparent after performing plaque assays. Virions were concentrated and partially purified using polyethylene glycol (PEG) precipitation and differential centrifugation. Atu_ph07 appeared to make small, turbid plaques on 0.3% soft agar (Figure 1A, left), and larger, clearer plaques on 0.15% soft agar (Figure 1A, right). Lowering the agar concentration allows propagation of jumbo phages by promoting phage diffusion through the medium. Growth curves of *A. tumefaciens* infected with Atu_ph07 at different multiplicities of infection (MOIs) show that Atu_ph07 inhibits growth of its host after 2 h (Figure 1B; Supplementary Figure S1A). Time-lapse microscopy of *A. tumefaciens* cells infected with Atu_ph07 at an MOI of 10 shows that Atu_ph07 causes cell lysis within 3 h (Figure 1C, Supplementary Movie M1). Transmission electron microscopy (TEM) of virions revealed icosahedral heads (length 146 ± 0.6 nm and width 152 ± 0.8 nm) and long tails (136 ± 0.5 nm), as shown in Figure 1D. Phage tails appear to be contractile, as shorter tails with an average length of 77 ± 2.6 nm were observed and some heads also appear to be empty. Lastly, the TEMs indicate the presence of tail fibers and “hairy” whiskers (Figure 1D, inset). Similar features have also been observed in Enterobacteria phage vB_PcaM_CBB (Buttimer et al., 2017a). While the “hairy” whiskers are tail-associated in Enterobacteria phage vB_PcaM_CBB (Buttimer et al., 2017a), these long, thin appendages appear to be primarily capsid-associated in Atu_ph07. Together, the morphology confirms that Atu_ph07 belongs to the family *Myoviridae* (Ackermann, 2009).

### Host range of Atu_ph07

**Figure 2:**
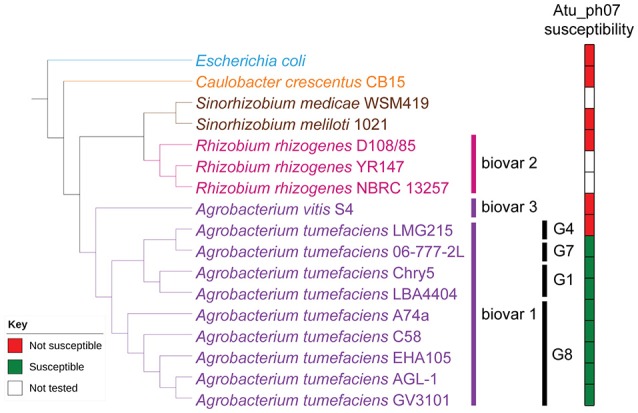
Host range of Atu_ph07. Phylogenetic tree of Alphaproteobacteria tested for Atu_ph07 susceptibility in this study was constructed using 16S rRNA sequences. *Agrobacterium* strains are represented in purple, *Rhizobium* strains in pink, *Sinorhizobium* strains in brown, *Caulobacter* in orange, and the outgroup, *Escherichia coli*, is in blue. Agrobacteria biovars and genomospecies are indicated. Phage susceptibility (green) or resistance (red) for each strain is indicated.

Since plaque formation by Atu_ph07 is inconsistent (Figure 1A), growth curves in the presence or absence of Atu_ph07 at an MOI of 10 were used to assess the host range of the phage (Supplementary Figure S1). Susceptible test strains have a decreased growth rate and growth yield in the presence of phage when compared to growth in the absence of phage (Figure 2, Supplementary Figure S1A), and plaques are formed when an undiluted phage stock with an infective titer of 7 × 10^10^ pfu/ml is spotted on the test strain. In contrast, resistant strains have comparable growth curves in the presence or absence of phage (Figure 2, Supplementary Figure S1B), and no plaques develop when the phage stock is spotted on lawns of the test strain.

To determine if similarity of the bacterial strains plays a role in phage infectivity, we acquired or sequenced the 16S rRNA gene of the host strains and constructed a phylogenetic tree (Figure 2). The strains of *A. tumefaciens* form a monophyletic clade consistent with the grouping of these closely related strains into Agrobacteria biovar 1 based on biochemical tests and pathogenicity assays (Keane et al., 1970; Panagopoulos and Psallidas, 1973). While it remains debated if biovar 1 comprises a single species (Sawada et al., 1993; Young et al., 2006) or a complex of related species (Mougel et al., 2002; Portier et al., 2006; Costechareyre et al., 2010), the bacterial strains in this group are heterogeneous, comprising at least nine genomospecies (G1–G9) (Mougel et al., 2002). *A. tumefaciens* C58, which was the host used to isolate Atu_ph07, belongs to the G8 genomospecies (Mougel et al., 2002) and each G8 strain tested was susceptible to Atu_ph07 (Figure 2). While other Agrobacteria biovar 1 strains belonging to G1 (LBA4404 and Chry5) are susceptible to Atu_ph07, this is not a universal phenotype as G4 strain LMG215 is resistant to Atu_ph07 (Figure 2). Representative isolates from biovars 2 and 3, as well as other Alphaproteobacterial strains, are not susceptible to Atu_ph07 infection (Figure 2). Thus, the host range of Atu_ph07 appears to be restricted within a subset of Agrobacteria biovar 1.

### Genome analysis and phylogeny

**Figure 3:**
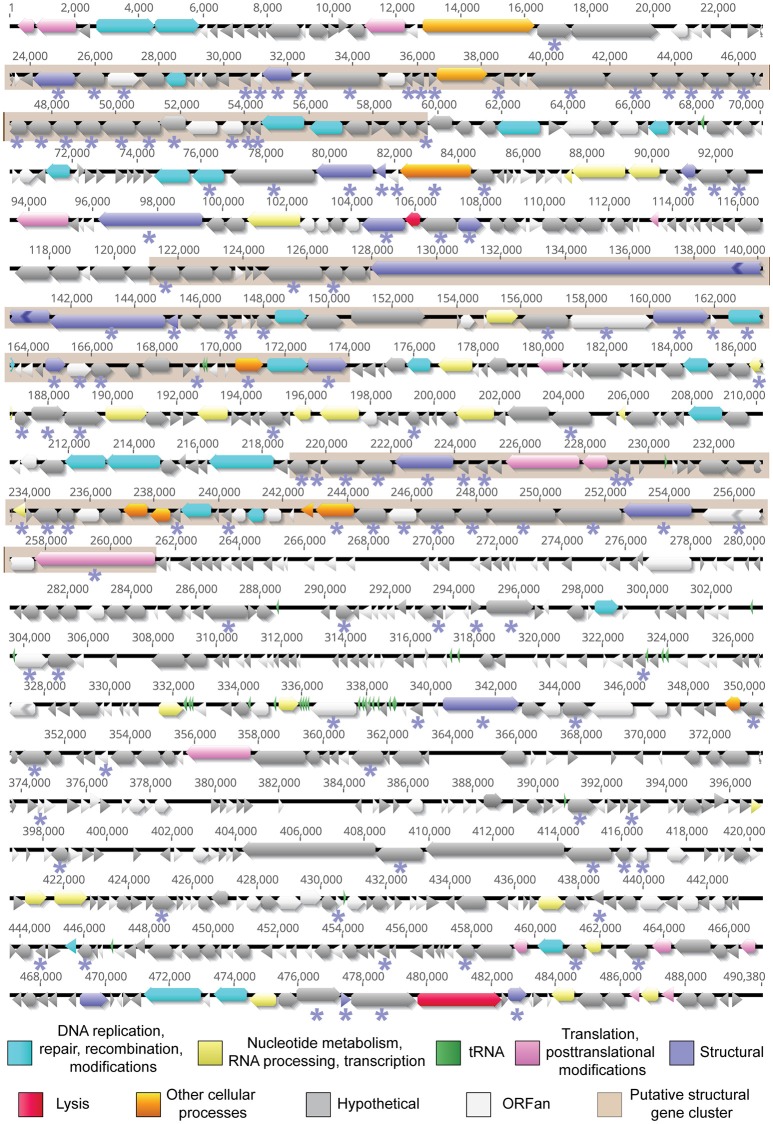
The annotated genome of Atu_ph07. ORFs are represented by functional categories in corresponding colors. Regions shaded in beige represent putative structural protein clusters as identified using ESI-MS/MS analysis. Proteins detected by ESI-MS/MS analysis are indicated with an asterisk (*) below the corresponding ORF.

The Atu_ph07 genome is 490,380 bp in length, leading to the classification of Atu_ph07 as a jumbo phage. Like other agriculturally-relevant jumbo phages, Atu_ph07 has a low G+C content (37.1%) (Almpanis et al., 2018). The genome was annotated using a combination of Rapid Annotation using Subsystem Technology (RAST) (Aziz et al., 2008) and manual annotation based on PSI-BLAST analysis (Altschul et al., 1997). Atu_ph07 contains 714 open reading frames (ORFs), including 390 ORFans (no discernable homologs in other lineages), 214 conserved hypothetical proteins with no assigned function, and 110 predicted proteins with assigned functions based on similarity to conserved proteins (Table 2, Supplementary Table S1, Figure 3).

**Table 2:** Summary of key genomic features of Atu_ph07.

**Genome length (bp)**	**G + C content (%)**	**Number of ORFs**	**Coding density (%)**	**Number of hypothetical proteins**	**Number of ORFs with predicted functions**	**Number of ORFans**	**Number of tRNAs**
490,380	37.1	714	83.6	214	110	390	33

**Figure 4:**
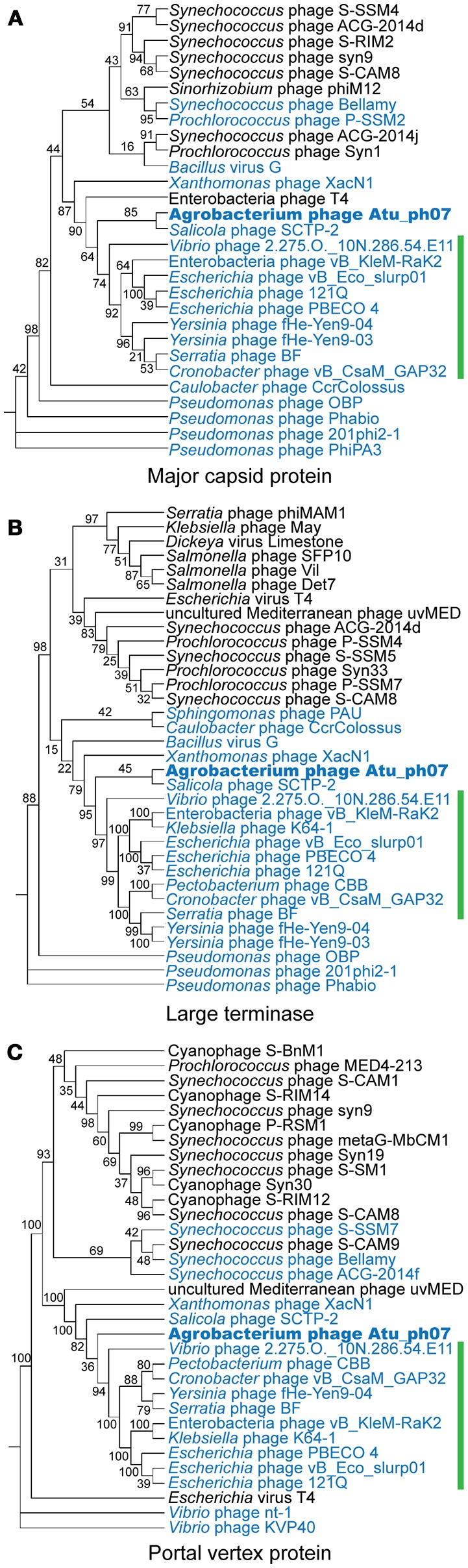
Phylogenetic comparison of Atu_ph07 and related phages. Phylogenetic trees of phages based on alignments of the **(A)** major capsid protein, **(B)** large terminase, and **(C)** portal vertex protein. Jumbo phages are labeled in blue and Atu_ph07 is indicated with bold font. Bootstrap values of 100 replicates are indicated. Rak2-like phages are indicated by a green line.

Due to the high degree of divergence, comparative genome analysis of jumbo phages is challenging. Based on nucleotide identity, Atu_ph07 is most similar to *Synechococcus* phage S-SSM7 (Sullivan et al., 2010). However, the genomes are only 13.1% identical and do not share collinear blocks. Since whole genome alignments did not reveal phages similar to Atu_ph07, we next constructed phylogenetic trees using the sequence of proteins conserved in many jumbo phages. There is no universal gene present in all phages, therefore signature gene products including the major capsid protein, large terminase subunit, and portal vertex protein were selected for the phylogenetic analysis (Adriaenssens and Cowan, 2014). These phylogenies place Atu_ph07 among the jumbo phages in the T4-superfamily (Figure 4). Although the genome of Atu_ph07 only shares 33 homologous ORFs with the genome of bacteriophage T4 (Supplementary Table S2), core proteins involved in phage morphogenesis and DNA replication, recombination, and repair were identified (Supplementary Table S3) (Miller et al., 2003). The phylogenetic trees are consistent with the recent characterization of *Xanthomonas* phage XacN1 (Yoshikawa et al., 2018), which suggested that XacN1, *Salicola* phage SCTP-2 and Atu_ph07 are distantly related to the Rak2-like jumbo phages.

**Figure 5:**
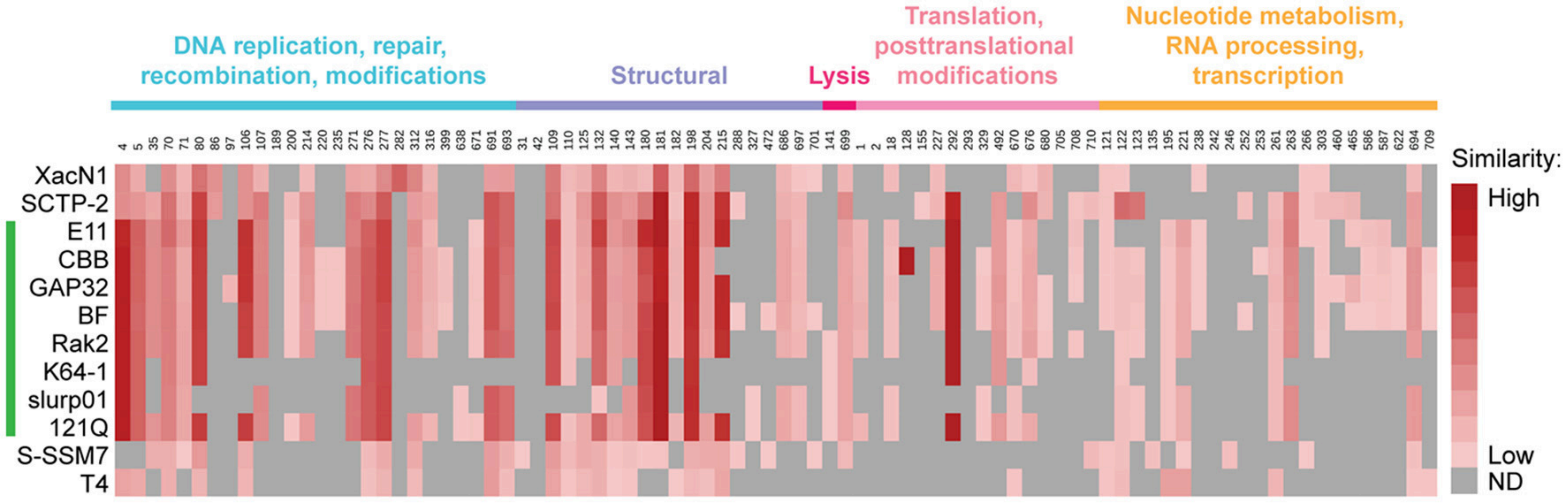
Similarity of annotated gene products in Atu_ph07 and related phages. Heat map displaying Atu_ph07 gene products compared with homologs in 12 related phages, including members of the Rak2-like phages (indicated with a green line). Intensity of the red color indicates the degree of similarity among homologs. Gray boxes indicate that a homolog with an E-value smaller than 1e-03 was not detected (ND). Gene products are organized by functional category and Atu_ph07 gp numbers are indicated.

The ORFs in the Atu_ph07 genome were compared to those in XacN1, SCTP-2, and Rak2-like phage genomes (Figure 5, Supplementary Table S2). The genome of phage SCTP-2 has the highest number of conserved genes with 141 genes (19.7% of Atu_ph07 ORFs) in common with the Atu_ph07 genome, perhaps due to the relatively large size of these phage genomes. Overall, the gene composition of Atu_ph07 is not well conserved with the Rak2-like phages (Figure 5, Supplementary Table S2), consistent with proposal that Atu_ph07, together with SCTP-2, may belong to a new clade that comprises a sister group to the Rak2-like phages (Yoshikawa et al., 2018).

### Functional annotation

Most of the 110 Atu_ph07 ORFs that can be assigned to a functional annotation are predicted to function in DNA replication, modification, recombination or repair (Figure 3, light blue arrows), nucleotide metabolism (Figure 3, yellow arrows), translation and posttranslational proteins (Figure 3, pink arrows) and structural proteins (Figure 3, purple arrows) (Supplementary Table S1).

#### DNA replication, repair, and recombination

Atu_ph07 encodes 26 enzymes involved in DNA replication, repair, and recombination (Figure 3, light blue arrows, Supplementary Table S1). The majority of these enzymes are all highly conserved in XacNI, SCTP-2, and the Rak2-like phages (Figure 5, Supplementary Table S2). These highly conserved enzymes include homologs of six of the T4 core proteins involved in DNA replication, repair, and recombination (Petrov et al., 2010). The enzymes with homology to the T4 core proteins are predicted to function as part of the DNA helicase-primase complex (gp70), DNA polymerases (gp276, gp277), sliding clamp loader (gp312), and recombination-related endonucleases (gp691, gp693) (Supplementary Table S3). One of the endonucleases (gp693) is directly upstream of a protein (gp694) with similarity to the RNA polymerase sigma factor for late transcription. This gene product shares similarity with corresponding proteins in XacNI, SCTP-2, T4, and most of the Rak2-like phages (Supplementary Table S2). The Atu_ph07 genome also encodes a predicted DNA polymerase III alpha subunit (gp316) and epsilon subunit (gp671) suggesting that the polymerase may contribute to both DNA replication and 3′-5′ exonuclease activity. The DNA polymerase III subunits are conserved in most of the Rak2-like phages (Figure 5, Supplementary Table S2). Atu_ph07 encodes three type II topoisomerase proteins involved in chromosome partitioning (gp4, gp5, gp200) (Kato et al., 1992). Gp4 is highly conserved with DNA gyrase subunit B encoded by the Rak2-like phages and gp5 encodes topoisomerase IV subunit A, also well-conserved in the Rak2-like phages (Figure 5). Together, the presence of these highly conserved genes suggests that Atu_ph07 encodes the proteins necessary to complete phage DNA replication and DNA-related functions including recombination and repair.

#### Nucleotide metabolism

To supplement the nucleotide pool required for phage DNA and RNA synthesis, T4-like phage genomes encode enzymes for nucleotide metabolism (Petrov et al., 2010). The Atu_ph07 genome contains several enzymes predicted to contribute to nucleotide metabolism (Figure 3, Supplementary Table S1). These include both alpha and beta subunits (gp122, gp123) of ribonucleotide reductase (RNR) of class 1a (*nrdA* and *nrdB*), which are involved in oxygen-dependent nucleotide metabolism of ribonucleotides into deoxyribonucleotides, a step that is needed for DNA replication (Dwivedi et al., 2013). RNR proteins catalyze nucleotide metabolism with the help of glutaredoxin and thioredoxin (Sengupta, 2014). Two putative glutaredoxin proteins are encoded by Atu_ph07 (gp121, gp266), one of which (gp121) is directly adjacent to the alpha subunit of RNR. Thioredoxin is encoded by gp221.

T4-like phages require ATP and NADH/NAD^+^ for important processes like DNA synthesis, transcription, and translation. To metabolize NAD^+^, phages use nicotinamide-adenine dinucleotide pyrophosphatase (NUDIX) hydrolases (Bessman et al., 1996; Lee et al., 2017). This family of enzymes is involved in housekeeping functions of the cell, including the hydrolysis of unwanted nucleotides or removal of excess metabolites. Atu_ph07 encodes three putative members of the NUDIX hydrolase superfamily (gp257, gp303, gp557). Other putative proteins involved in nucleotide metabolism include NadR (gp587) and PnuC (gp586). NadR transcriptionally regulates NAD biosynthesis and PnuC is a membrane transporter that allows nicotinamide mononucleotide (NMN) uptake (Foster et al., 1990; Kurnasov et al., 2002).

#### tRNA genes and tRNA processing genes

**Figure 6:**
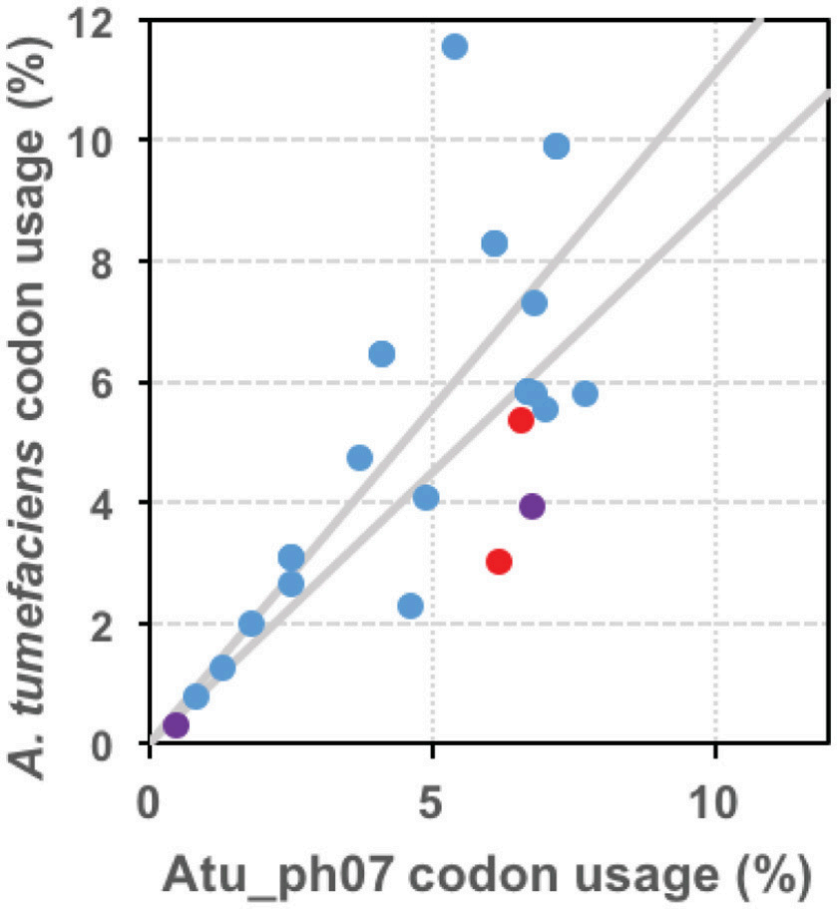
tRNAs are encoded in the Atu_ph07 genome. Graphical representation of codon bias of phage Atu_ph07 and its host *A. tumefaciens* strain C58. Data points represent the usage of each codon in the Atu_ph07 and *A. tumefaciens* genomes. Red points represent codons only found in *A. tumefaciens*, purple points represent codons only found in Atu_ph07, and blue points represent codons found in both genomes. Gray lines outline the region in which codon usage in both genomes is similar.

The genome of Atu_ph07 encodes 33 tRNA genes, including 32 canonical tRNAs corresponding to all amino acids except asparagine and threonine (Figure 6, Supplementary Table S4). The remaining tRNA is a suppressor with an anticodon of UCA indicating read-through of opal (UGA) stop codons. The UGA stop codon is abundant in both the phage (*N* = 267) and *A. tumefaciens* (*N* = 2,923) genomes suggesting that there are several potential genes targeted by the suppressor tRNA. With the exception of the suppressor tRNA, all of the tRNAs encoded in the Atu_ph07 genome are also found in the *A. tumefaciens* genome suggesting that the tRNAs do not improve decoding capacity; however, some of the phage tRNAs correspond to codons that are more frequently used in the phage genome (Figure 6). This observation is consistent with the notion that phage-encoded tRNAs allow translation to be optimized for the codon usage of the phage genome (Bailly-Bechet et al., 2007). In addition to the tRNA genes, the Atu_ph07 genome encodes four tRNA processing proteins. These tRNA processing proteins include tRNA nucleotidyltransferase (gp18) and tRNA^His^-5′-guanylyltransferase (gp227), which are involved in tRNA maturation. Putative peptidyl-tRNA hydrolases (gp680, gp256) function to decrease the pool of peptidyl-tRNAs formed throughout the initiation, elongation, and termination stages of translation.

#### Clp-like proteins

The Atu_ph07 phage genome encodes seven putative members of the Clp family of proteins that function to degrade proteins, including ClpX (gp2), a prophage Clp protease-like protein (gp128), an ATP-dependent Clp protease ATP-binding subunit (gp155), ClpA (gp292), ClpB (gp492), ATP-dependent Clp protease proteolytic subunit (gp676), and ClpS (gp708). The Clp proteases may contribute to virion assembly or have alternative functions. For example, during phage lambda DNA replication, the ClpX/ClpP protease removes the O protein from the origin of replication (Zylicz et al., 1998) and the activity of ClpX/ClpP has been associated with slowing down DNA replication of the phage under poor growth conditions (Wegrzyn et al., 2000). In the Atu_ph07 genome, *clpX* (gp2) is located in close proximity to genes predicted to encode topoisomerase proteins (gp4-5) suggesting that the ClpX protein may function in the regulation of phage DNA replication. In *E. coli*, ClpS is an adaptor protein that modifies the substrate specificity of the ClpA/ClpP protease and contributes to degradation or refolding of protein aggregates (Dougan et al., 2002). ClpA (gp292) is highly conserved in most of the Rak2-like phages (Figure 5). The presence of the ClpB (gp492) and DnaJ (gp293) chaperones, which also function in the removal of protein aggregates (Mogk et al., 1999), further suggests that Atu_ph07 may help its host to survive the stress of phage infection long enough for the phage replication cycle to be completed. Together, these observations suggest that Clp proteins likely contribute to diverse aspects of phage biology potentially including virion assembly, DNA replication, and proteolytic clearance of protein aggregates.

### Structural proteins

Based on homology, the genome of Atu_ph07 was predicted to encode 20 proteins involved in phage morphogenesis and structure (Figure 3, Supplementary Table S1). Candidate structural genes encode proteins for head morphogenesis and structure (gp125, gp143, gp198, gp204, gp215), baseplate (gp181, gp182, gp686, gp697), tail sheath (gp132, gp140), and tail fibers (gp31, gp288, gp327, gp472). All five of the candidate head proteins, one of the baseplate wedge subunits (gp182), and both of the tail sheath proteins share significant homology with T4 core structural proteins (Supplementary Tables S2, S3). The Atu_ph07 genome does not encode proteins with similarity to T4 core tail fiber proteins (Supplementary Table S3); however short tail fibers are evident when Atu_ph07 is observed under transmission electron microscopy (Figure 1D). One of the tail fiber proteins (gp472) is most closely related (51% identity) to a tail fiber protein encoded in the genome of *Agrobacterium* phage Atu_ph02 (Attai et al., 2017), suggesting that these phages may share an entry route into *Agrobacterium* cells.

**Figure 7:**
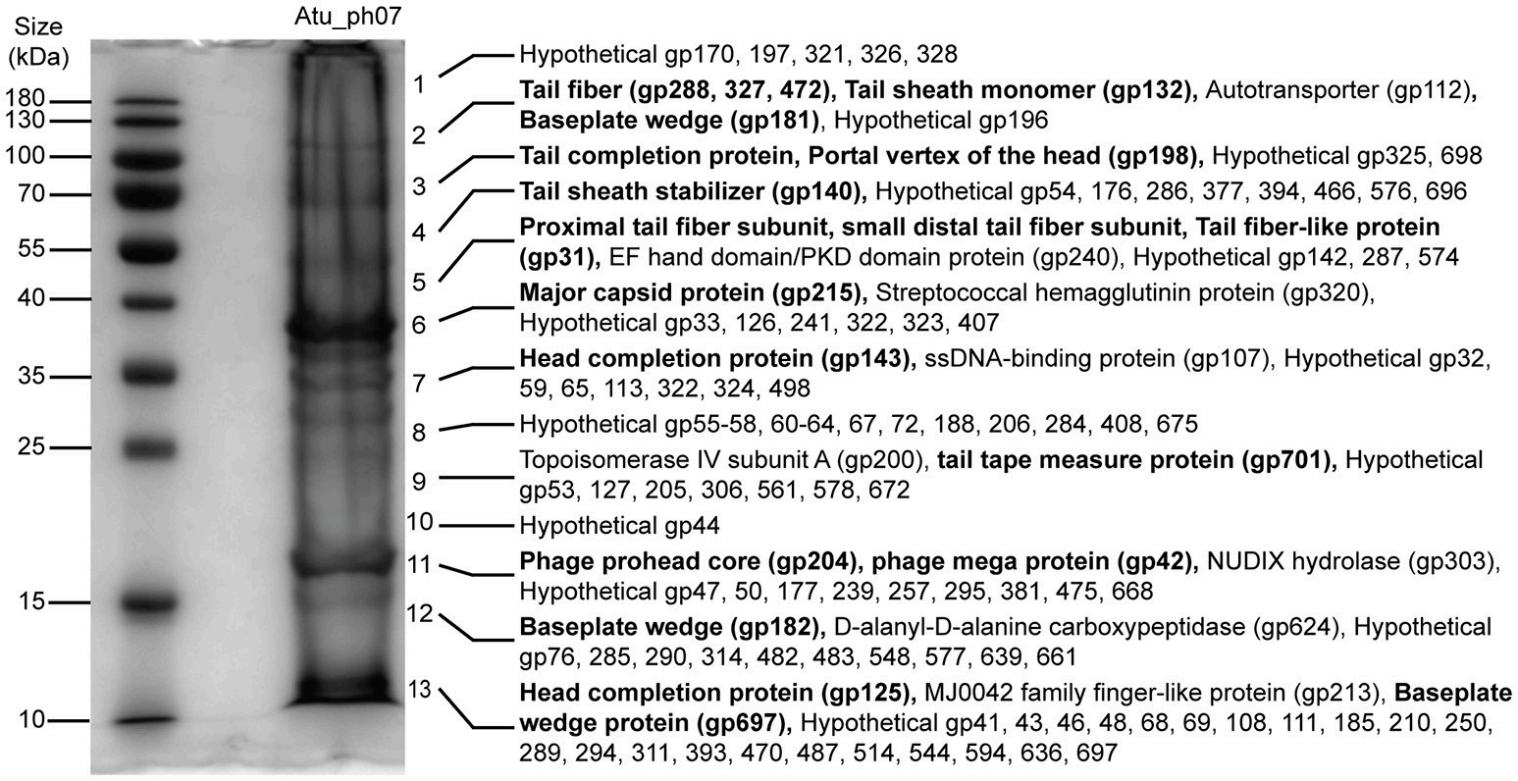
SDS-PAGE of Atu_ph07 structural proteins as identified by ESI-MS/MS. Phage proteins were separated by size and fragments covering the full lane of the gel were excised for proteomic analysis. Numbers at the right of the gel indicate the position of fragments which were excised from the gel. Proteins identified in each fragment are listed. Bold font indicates validation of annotated structural proteins.

Based upon the complex morphology of Atu_ph07, we hypothesized that the genome annotation likely underestimates the quantity of proteins involved in phage morphogenesis and structure. To experimentally identify structural proteins, electrospray ionization mass spectrometry (ESI-MS/MS) was used (Figure 7, Supplementary Table S5). Indeed, the number of structural proteins identified by ESI-MS/MS (indicated by purple asterisks in Figure 3) is greater than the number of structural proteins predicted by the genome annotation (purple arrows in Figure 3). Overall, the proteomic analysis supported the annotation of structural proteins, as all of the head, neck, tail fiber, and most of the tail (5/7) proteins could be identified in the virion proteome. As expected, the most abundant protein observed in the particle proteome is the major capsid protein (gp215, fragment 6 in Figure 7). A total of 131 proteins were found among the phage virion proteins, with sequence coverages higher than 5%, or more than one identified unique peptide (Supplementary Table S5). About 78% (102/131) of these proteins do not have an assigned function. The majority of the virion proteins are encoded in three large clusters CDS 31-76, CDS 170-215, and CDS 284-329 (shaded in beige in Figure 3) that contain a high proportion of structural proteins. The remaining proteins reside in smaller clusters or as separate genes spread across the genome. Notably, CDS 31-76 contains 29 identified proteins of which only two were predicted as structural proteins in the genome, indicative of the presence of unique structural proteins in Atu_ph07. The lack of similarity with known virion proteins should not be surprising, as only a small number of jumbo phages with whisker-like structures have been described to date.

### Atu_ph07 induces cell lysis

The genome content is insufficient to confidently predict the phage life cycle, however the phage can induce lysis (Figures 1B,C). To assess the possibility of a lysogenic phase, we isolated ten variant clones of *A. tumefaciens* strain C58 that survive exposure to Atu_ph07 at high MOI. Lysogens were not induced from any of the 10 unsusceptible variant clones by exposing them to UV irradiation or mitomycin C (see Materials and Methods for experimental details). Furthermore, we were unable to PCR-amplify two distinctive Atu_ph07 genes, nicotinate phosphoribosyltransferase (CDS 242), and adenine-specific methyltransferase (CDS 399), from the unsusceptible variants. Consistent with these observations, no integrase- or Cro-like genes, which are required for lysogeny in several temperate phage species, could be identified in the Atu_ph07 genome.

Since we observe that Atu_ph07 can induce cell lysis (Figure 1C), we searched for genes encoding candidate lysis proteins in the genome. Atu_ph07 contains two predicted lysozymes (gp141 and gp699). Gp141 is in close proximity to the predicted phage head completion protein and tail sheath monomer, indicating that it may be involved in phage DNA entry. Gp699 has homologes in the Rak2-like phages and is adjacent to three predicted structural proteins—the baseplate hub subunit (gp686), baseplate wedge protein (gp697), and tail tape measure protein (gp701).

To widen our search for candidate lysis proteins, we searched for transmembrane (TM) proteins which can be indicative of the presence of the canonical endolysin-holin-spanin system of host cell lysis (Young, 2014). A TMHMM analysis (Krogh et al., 2001) of the predicted proteins identified 40 predicted TM proteins, only three of which have putative functions: ribonucleotide reductase of class 1a beta subunit (gp123), ribosyl nicotinamide transporter PnuC (gp586), and peptidyl tRNA hydrolase (gp680). Eight of these encoded TM proteins (gp369-gp376) appear consecutively on the genome at ~280 kbp, however each of these are ORFs with no detectable similarity in the database, or ORFans (Yin and Fischer, 2008), and therefore we were unable to predict their function as a unit at this time. Thus, at present, the mechanism of Atu_ph07-mediated host cell lysis remains unknown.

## Conclusion

Several jumbo phages have been recently characterized, many encoding a large number of hypothetical proteins. Recently, a group of T4-like phages have been categorized into a new monophyletic group called “Rak2-like.” While phage Atu_ph07 clusters just outside this group, many genes share homology with core genes in the Rak2-like phage genomes. Atu_ph07 infects a subset of *A. tumefaciens* strains (Figure 2) and its ability to infect this plant pathogen makes it a candidate for biocontrol.

The phage biology of Atu_ph07 is likely to be remarkable in its own right. While the genome encodes genes for DNA replication, transcription, translation, nucleotide metabolism, as well as over 130 experimentally confirmed structural proteins, many more molecular mechanisms remain to be unraveled. Understanding the modes through which a non-living entity can acquire, store, replicate, and express such a vast number of genes to promote its life cycle is a fascinating aspect of phage biology unique to jumbo phages. A logical assumption considering the coding density of this phage is that many of the putative hypothetical proteins have functional significance and provide jumbo phages with an evolutionary advantage in specific ecological niches. Continued exploration of jumbo phages will help elucidate the mechanisms in which diverse bacteriophages have evolved to thrive as the most abundant biological entities in the world.

## Author contributions

HA, MB, KP, and J-PN conducted experiments. All authors designed experiments and analyzed data. HA, MB, RL, and PB contributed to writing and editing of the manuscript.

### Conflict of interest statement

The authors declare that the research was conducted in the absence of any commercial or financial relationships that could be construed as a potential conflict of interest.
