# Supplementary material for: Larger Than Life: Isolation and Genomic Characterization of a Jumbo Phage That Infects the Bacterial Plant Pathogen, Agrobacterium tumefaciens
Source: Front Microbiol. 2018 Aug 14;9:1861. doi: 10.3389/fmicb.2018.01861 (PMC6102473; doi:10.3389/fmicb.2018.01861)
Supplement: Supplementary file 2 [file Data_Sheet_1.docx]

***Supplementary Material:***

**Larger than Life: Isolation and genomic characterization of a jumbo phage that infects the bacterial plant pathogen, *Agrobacterium tumefaciens***

Hedieh Attai, Maarten Boon, Kenya Phillips, Jean-Paul Noben, Rob Lavigne, Pamela J. B. Brown*

*Correspondence:

Pamela J. B. Brown

brownpb@missouri.edu

SUPPLEMENTARY MOVIES

**Supplementary Movie M1.** Time-lapse microscopy of *Agrobacterium tumefaciens* strain C58 infected with Atu_ph07 at an MOI of 10 for 180 minutes. Movie taken at 5 fps.

SUPPLEMENTARY FIGURES

**Supplementary Figure S1.** Representative growth curves of *A. tumefaciens* strains C58 (A) and LMG215 (B) in the absence (blue) or presence (red) of phage Atu_ph07 at MOI 10. Experiment was performed in duplicate and averaged.

**A B**

SUPPLEMENTARY TABLES

**Supplementary Table S1.** Atu_ph07 genes categorized by predicted function.

| **CDS #** | **RAST assigned function** | **Updated assigned function** | **Length (bp)** | **Category** |
| --- | --- | --- | --- | --- |
| 1 | Translation initiation factor 3 CDS |  | 504 | Translation |
| 2 | ATP-dependent Clp protease ATP-binding subunit ClpX CDS |  | 1,260 | Posttranslational modification |
| 4 | Phage DNA topoisomerase large subunit (EC 5.99.1.3) #T4-like gp60 #T4 GC1464 CDS | DNA gyrase subunit B | 1,815 | DNA replication |
| 5 | Topoisomerase IV subunit A (EC 5.99.1.-) CDS |  | 1,362 | DNA replication |
| 18 | tRNA nucleotidyltransferase (EC 2.7.7.21) (EC 2.7.7.25) CDS |  | 1,266 | Translation |
| 20 | internalin, putative CDS |  | 3,507 | Other |
| 31 | Hypothetical protein CDS | Phage tail fiber-like protein | 1,290 | Structural |
| 35 | Hypothetical protein CDS | Exonuclease type II | 681 | DNA replication |
| 42 | conserved phage mega protein |  | 921 | Structural |
| 49 | multi-sensor signal transduction histidine kinase CDS |  | 1,611 | Other |
| 70 | DNA primase/helicase, phage-associated CDS |  | 1,323 | DNA replication |
| 71 | Hypothetical protein CDS | DNA primase subunit | 1,023 | DNA replication |
| 80 | Hypothetical protein CDS | RNA-DNA + DNA-DNA helicase | 1,383 | DNA replication |
| 86 | Hypothetical protein CDS | 2'-5' RNA ligase | 645 | DNA replication |
| tRNA | tRNA-Gln-TTG |  | 125 | tRNA |
| 97 | putative type II DNA modification enzyme (methyltransferase) CDS |  | 768 | DNA modification |
| 106 | Hypothetical protein CDS | RecA-like protein | 1,107 | DNA recombination and repair |
| 107 | Hypothetical protein CDS | ssDNA-binding protein | 1002 | DNA replication |
| 109 | Gp17 terminase DNA packaging enzyme large subunit CDS |  | 1,809 | Structural |
| 110 | Phage terminase, large subunit CDS |  | 372 | Structural |
| 112 | autotransporter CDS |  | 2,235 | Other |
| 121 | Hypothetical protein CDS | Glutaredoxin | 237 | Nucleotide metabolism |
| 122 | Ribonucleotide reductase of class Ia (aerobic), alpha subunit (EC 1.17.4.1) CDS |  | 1,641 | Nucleotide metabolism |
| 123 | Ribonucleotide reductase of class Ia (aerobic), beta subunit (EC 1.17.4.1) CDS |  | 999 | Nucleotide metabolism |
| 125 | Phage head completion protein CDS |  | 483 | Structural |
| 128 | Prophage Clp protease-like protein CDS |  | 1,656 | Posttranslational modification |
| 132 | Phage tail sheath monomer CDS |  | 3,261 | Structural |
| 135 | NAD synthetase (EC 6.3.1.5) / Glutamine amidotransferase chain of NAD synthetase CDS |  | 1,671 | Nucleotide metabolism |
| 140 | Hypothetical protein CDS | Tail sheath stabilizer and completion protein | 1,374 | Structural |
| 141 | Phage lysin (EC 3.2.1.17) # Phage lysozyme or muramidase (EC 3.2.1.17) CDS |  | 450 | Lysis |
| 143 | Hypothetical protein CDS | Head completion adaptor; neck | 771 | Structural |
| 155 | Hypothetical protein CDS | ATP-dependent Clp protease ATP-binding subunit | 285 | Posttranslational modification |
| 180 | T4-like phage structural protein CDS |  | 13,350 | Structural |
| 181 | Hypothetical protein CDS | Baseplate wedge | 3,597 | Structural |
| 182 | Hypothetical protein CDS | Baseplate wedge | 390 | Structural |
| 189 | ulcer associated adenine specific DNA methyltransferase CDS |  | 978 | DNA modification |
| 191 | Phage protein CDS |  | 2,289 | Hypothetical |
| 195 | T4-like phage protein, T4 GC1542 CDS | Nucleotidyltransferase | 993 | Nucleotide metabolism |
| 198 | Phage portal vertex of the head #T4-like phage Gp20 CDS |  | 1,755 | Structural |
| 200 | Topoisomerase IV subunit A (EC 5.99.1.-) CDS |  | 1,170 | DNA replication |
| 204 | Phage prohead core protein CDS - Gp21 |  | 633 | Structural |
| 208 | Phage protein CDS |  | 819 | Hypothetical |
| tRNA | tRNA-Leu-CAA |  | 78 | tRNA |
| tRNA | tRNA-Met-CAT |  | 76 | tRNA |
| 213 | MJ0042 family finger-like protein CDS |  | 876 | Other |
| 214 | DNA double-strand break repair Rad50 ATPase CDS |  | 1,245 | DNA recombination and repair |
| 215 | Phage major capsid protein of Caudovirales (T4-like gp23) CDS |  | 1,230 | Structural |
| 219 | Conserved protein CDS |  | 651 | Hypothetical |
| 220 | Adenine-specific methyltransferase (EC 2.1.1.72) CDS |  | 783 | DNA modification |
| 221 | Thioredoxin, phage-associated CDS |  | 1,047 | Nucleotide metabolism |
| 227 | tRNAHis-5'-guanylyltransferase CDS |  | 789 | Translation |
| 235 | Adenine-specific methyltransferase (EC 2.1.1.72) CDS |  | 810 | DNA modification |
| 238 | ADP-ribose pyrophosphatase (EC 3.6.1.13) CDS |  | 498 | Nucleotide metabolism |
| 240 | EF hand domain/PKD domain protein CDS |  | 1,017 | Hypothetical |
| 242 | Nicotinate phosphoribosyltransferase (EC 2.4.2.11) CDS |  | 1,299 | Nucleotide metabolism |
| 246 | Thymidylate synthase thyX (EC 2.1.1.-) CDS |  | 951 | Nucleotide metabolism |
| 252 | HNH endonuclease family protein CDS |  | 558 | Nucleotide metabolism |
| 253 | protein serine-threonine phosphatase CDS |  | 1,257 | Transcription |
| 261 | RNA ligase, phage-associated #T4-like RnlA #T4 GC1653 CDS |  | 1,194 | Transcription |
| 263 | Sll7028 protein CDS | Zn dependent hydrolase | 1,290 | Nucleotide metabolism |
| 266 | Glutaredoxin CDS |  | 258 | Nucleotide metabolism |
| 271 | DNA polymerase I (EC 2.7.7.7) CDS |  | 1,062 | DNA replication |
| 276 | DNA polymerase (EC 2.7.7.7), phage-associated #T4-like phage gp43 #T4 GC0178 CDS |  | 1,254 | DNA replication |
| 277 | DNA polymerase (EC 2.7.7.7), phage-associated CDS |  | 1,698 | DNA replication |
| 279 | Phage protein CDS |  | 249 | Hypothetical |
| 282 | DNA ligase (EC 6.5.1.2) CDS |  | 1,995 | DNA replication |
| 288 | Phage tail fiber protein CDS |  | 1,833 | Structural |
| 292 | ATP-dependent Clp protease ATP-binding subunit ClpA CDS |  | 2,298 | Posttranslational modification |
| 293 | Chaperone protein DnaJ CDS |  | 807 | Posttranslational modification |
| tRNA | tRNA Ser-GCT |  | 93 | tRNA |
| 303 | Probable NUDIX hydrolase CDS |  | 393 | Nucleotide metabolism |
| 309 | Phosphate starvation-inducible protein PhoH, predicted ATPase CDS |  | 765 | Other |
| 310 | Hypothetical protein CDS | Phosphoglycolate phosphatase | 621 | Other |
| 312 | Replication factor C small subunit CDS |  | 948 | DNA replication |
| 316 | Hypothetical protein CDS | DNA polymerase III alpha subunit | 534 | DNA replication |
| 319 | Cyanophage-encoded pyrophosphatase, MazG #T4 GC0184 CDS |  | 408 | Other |
| 320 | Streptococcal hemagglutinin protein CDS |  | 1,182 | Other |
| 327 | Phage tail fiber protein CDS |  | 2,130 | Structural |
| 329 | Extracellular serine proteinase precursor (EC 3.4.21.-) CDS |  | 3,756 | Posttranslational modification |
| tRNA | tRNA-Pseudo-ACC |  | 74 | tRNA |
| 297 | Conserved protein CDS |  | 297 | Hypothetical |
| 393 | Phage protein CDS |  | 312 | Hypothetical |
| 394 | no significant homology |  | 1,455 | Hypothetical |
| 399 | Adenine-specific methyltransferase (EC 2.1.1.72) CDS |  | 759 | DNA modification |
| tRNA | tRNA-Gly-GCC |  | 75 | tRNA |
| tRNA | tRNA-Met-CAT |  | 78 | tRNA |
| tRNA | tRNA-Asp-GTC |  | 78 | tRNA |
| tRNA | tRNA-Arg-TCT |  | 78 | tRNA |
| tRNA | tRNA-His-GTG |  | 75 | tRNA |
| tRNA | tRNA-Ser-TGA |  | 92 | tRNA |
| tRNA | tRNA-Tyr-GTA |  | 119 | tRNA |
| tRNA | tRNA-Leu-TAG |  | 90 | tRNA |
| tRNA | tRNA-Gly-TCC |  | 72 | tRNA |
| tRNA | tRNA-Gln-TTG |  | 75 | tRNA |
| tRNA | tRNA-Pro-TGG |  | 77 | tRNA |
| tRNA | tRNA-Ser-TGA |  | 109 | tRNA |
| 460 | Hypothetical protein CDS | HNH endonuclease | 765 | Nucleotide metabolism |
| 465 | Phage-associated homing endonuclease CDS |  | 609 | Nucleotide metabolism |
| tRNA | tRNA-Phe-GAA |  | 76 | tRNA |
| tRNA | tRNA-Gln-CTG |  | 74 | tRNA |
| tRNA | tRNA-Pro-TGG |  | 78 | tRNA |
| tRNA | tRNA-Val-TAC |  | 75 | tRNA |
| tRNA | tRNA-Glu-TTC |  | 77 | tRNA |
| tRNA | tRNA-Met-CAT |  | 72 | tRNA |
| tRNA | tRNA-Trp-CCA |  | 76 | tRNA |
| tRNA | tRNA-Tyr-GTA |  | 82 | tRNA |
| tRNA | tRNA-Ile-GAT |  | 80 | tRNA |
| tRNA | tRNA-Pro-TGG |  | 78 | tRNA |
| tRNA | tRNA-Phe-GAA |  | 76 | tRNA |
| tRNA | tRNA-Arg-ACG |  | 78 | tRNA |
| tRNA | tRNA-Ala-TGC |  | 72 | tRNA |
| 472 | Phage tail fiber protein CDS |  | 2,361 | Structural |
| 481 | Guanosine-3',5'-bis(Diphosphate) 3'-pyrophosphohydrolase (EC 3.1.7.2) CDS |  | 456 | Nucleotide metabolism |
| 492 | ClpB protein CDS |  | 1,986 | Posttranslational modification |
| tRNA | tRNA-Lys-CCT |  | 73 | tRNA |
| 554 | Conserved protein CDS |  | 384 | Hypothetical |
| 557 | Hypothetical protein CDS |  | 306 | Hypothetical |
| 573 | Autotransporter adhesin CDS | Hypothetical protein | 4,152 | Hypothetical |
| 575 | High-affnity carbon uptake protein Hat/HatR CDS |  | 4,317 | Hypothetical |
| 586 | Ribosyl nicotinamide transporter, PnuC-like CDS |  | 678 | Nucleotide metabolism |
| 587 | Nicotinamide-nucleotide adenylyltransferase, NadR family (EC 2.7.7.1) / Ribosylnicotinamide kinase (EC 2.7.1.22) CDS |  | 1,026 | Nucleotide metabolism |
| tRNA | tRNA-Sup-TCA |  | 72 | tRNA |
| 622 | Hypothetical protein CDS | HNH endonuclease | 789 | Nucleotide metabolism |
| 624 | Putative phage-related protein precursor CDS | D-alanyl-D-alanine carboxypeptidase | 399 | Other |
| 638 | FIG006762: Phosphoglycerate mutase family CDS | RNAse HI | 369 | DNA replication |
| tRNA | tRNA-Cys-GCA |  | 76 | tRNA |
| 670 | ADP-ribose 1""-phosphate phosphatase related protein CDS |  | 438 | Posttranslational modification |
| 671 | Polymerase epsilon subunit CDS |  | 792 | DNA replication |
| 673 | Uncharacterized protein COG3236 CDS |  | 537 | Hypothetical |
| 676 | ATP-dependent Clp protease proteolytic subunit (EC 3.4.21.92) CDS |  | 615 | Posttranslational modification |
| 677 | Phage protein CDS |  | 1,125 | Hypothetical |
| 680 | Peptidyl-tRNA hydrolase, archaeal type (EC 3.1.1.29) CDS |  | 468 | Translation |
| 686 | Hypothetical protein CDS | Baseplate hub subunit | 843 | Structural |
| 691 | Phage recombination-related endonuclease Gp46 CDS |  | 1,776 | DNA recombination and repair |
| 693 | Phage recombination related endonuclease CDS |  | 1,023 | DNA recombination and repair |
| 694 | Hypothetical protein CDS | RNA polymerase sigma factor | 834 | Transcription |
| 697 | Hypothetical protein CDS | Baseplate wedge | 315 | Structural |
| 699 | Hypothetical protein CDS | Lysozyme | 2,598 | Lysis |
| 701 | PE-PGRS virulence associated protein CDS | Tail tape measure protein | 618 | Structural |
| 705 | Threonyl-tRNA synthetase (EC 6.1.1.3) CDS |  | 708 | Translation |
| 708 | ATP-dependent Clp protease adaptor protein ClpS CDS |  | 303 | Posttranslational modification |
| 709 | Thymidine kinase (EC 2.7.1.21) CDS |  | 549 | Nucleotide metabolism |
| 710 | 16 kDa heat shock protein A CDS | Molecular chaperone IbpA, HSP20 family | 402 | Posttranslational modification |

**Supplementary Table S2.** Atu_ph07 gene products compared with 12 related phages.

| **gp** | **XacNI** | **SCTP-2** | **BF** | **CBB** | **GAP32** | **121Q** | **K64-1** | **E11** | **Rak2** | **slurp01** | **S-SSM7** | **T4** |
| --- | --- | --- | --- | --- | --- | --- | --- | --- | --- | --- | --- | --- |
| DNA replication, repair, recombination | | | | | | | | | | | | |
| 4 | 1.00E-65 | 1.00E-62 | 1.00E-179 | 4.00E-176 | 5.00E-179 | 6.00E-177 | 2.00E-177 | 2.00E-167 | 4.00E-177 | 2.00E-178 |  | 3.00E-41 |
| 5 | 1.00E-34 | 4.00E-53 | 3.00E-101 | 4.00E-107 | 4.00E-107 | 5.00E-107 | 2.00E-107 | 3.00E-109 | 4.00E-107 | 9.00E-107 |  | 5.00E-36 |
| 35 |  | 3.00E-37 | 1.00E-65 | 8.00E-66 | 2.00E-65 | 5.00E-58 |  | 2.00E-63 | 3.00E-63 |  | 6.00E-31 |  |
| 70 | 6.00E-64 | 4.00E-90 | 2.00E-79 | 1.00E-76 | 2.00E-77 | 4.00E-75 | 8.00E-77 | 7.00E-96 | 8.00E-77 | 2.00E-75 | 4.00E-31 | 4.00E-29 |
| 71 | 2.00E-24 | 7.00E-31 | 9.00E-40 | 1.00E-36 | 4.00E-35 | 8.00E-38 | 1.00E-42 | 7.00E-52 | 3.00E-42 | 5.00E-38 | 1.00E-04 |  |
| 80 | 2.00E-91 | 9.00E-138 | 9.00E-146 | 1.00E-147 | 3.00E-146 | 2.00E-145 | 5.00E-141 | 2.00E-151 | 7.00E-141 |  | 6.00E-51 | 1.00E-26 |
| 86 | 1.00E-59 | 2.00E-52 |  |  |  |  |  |  |  |  |  |  |
| 97 |  |  |  |  | 2.00E-24 |  |  |  |  |  |  |  |
| 106 | 2.00E-60 | 6.00E-59 | 2.00E-141 | 8.00E-154 | 5.00E-153 | 6.00E-143 |  | 2.00E-155 | 3.00E-140 |  | 2.00E-31 | 7.00E-24 |
| 107 | 2.00E-28 | 4.00E-81 | 7.00E-74 | 2.00E-70 | 9.00E-74 | 1.00E-66 |  | 8.00E-71 | 6.00E-73 | 8.00E-67 |  |  |
| 189 |  |  |  |  |  |  |  |  |  |  |  |  |
| 200 |  | 5.00E-15 | 5.00E-06 | 8.00E-06 | 4.00E-05 | 5.00E-05 |  | 4.00E-06 | 5.00E-07 |  |  |  |
| 214 | 2.00E-18 | 7.00E-55 | 4.00E-48 | 4.00E-53 | 6.00E-51 | 3.00E-49 |  | 2.00E-62 | 3.00E-50 |  |  |  |
| 220 |  |  | 1.00E-09 | 5.00E-09 | 6.00E-09 |  |  |  |  |  |  |  |
| 235 |  |  | 4.00E-09 | 4.00E-09 | 3.00E-09 |  |  |  |  |  |  |  |
| 271 | 1.00E-39 | 6.00E-80 | 5.00E-86 | 2.00E-85 | 1.00E-83 | 2.00E-87 |  | 2.00E-75 | 1.00E-87 | 1.00E-87 |  |  |
| 276 | 5.00E-36 | 6.00E-67 | 2.00E-116 | 3.00E-115 | 6.00E-116 | 7.00E-116 | 8.00E-118 | 3.00E-109 | 5.00E-117 | 7.00E-116 | 4.00E-15 | 2.00E-11 |
| 277 | 5.00E-68 | 2.00E-116 | 2.00E-133 | 5.00E-137 | 9.00E-139 | 1.00E-135 | 8.00E-133 | 2.00E-137 | 6.00E-133 | 2.00E-135 | 6.00E-33 | 2.00E-26 |
| 282 | 1.00E-108 |  |  |  |  |  |  |  |  |  |  |  |
| 312 | 4.00E-70 | 5.00E-48 | 4.00E-49 | 4.00E-51 | 3.00E-52 | 2.00E-47 |  | 3.00E-61 | 4.00E-46 |  | 5.00E-33 | 3.00E-18 |
| 316 | 6.00E-25 | 1.00E-37 | 1.00E-26 | 1.00E-27 | 2.00E-27 | 3.00E-24 |  | 5.00E-22 | 5.00E-26 |  |  |  |
| 399 |  |  | 1.00E-09 | 1.00E-11 | 3.00E-13 |  |  |  |  |  |  |  |
| 638 |  |  |  |  |  | 2.00E-05 |  |  |  | 4.00E-06 |  |  |
| 671 |  |  | 9.00E-08 | 5.00E-08 | 8.00E-09 | 4.00E-09 |  | 0.001 | 3.00E-07 |  |  |  |
| 691 | 7.00E-48 | 1.00E-120 | 6.00E-124 | 8.00E-124 | 1.00E-125 | 3.00E-113 |  | 3.00E-119 | 1.00E-109 | 6.00E-113 | 5.00E-48 | 7.00E-50 |
| 693 | 2.00E-49 | 1.00E-100 | 1.00E-91 | 2.00E-92 | 4.00E-91 | 2.00E-93 |  | 2.00E-101 | 2.00E-96 | 1.00E-93 | 6.00E-27 | 8.00E-15 |
| Structural | | | | | | | | | | | | |
| 31 |  |  |  |  |  |  |  |  |  |  | 2.00E-04 |  |
| 42 |  |  |  |  |  |  |  |  |  |  |  |  |
| 109 | 2.00E-83 | 1.00E-91 | 4.00E-134 | 1.00E-130 | 3.00E-131 | 3.00E-130 | 2.00E-122 | 7.00E-133 | 4.00E-123 |  | 9.00E-49 | 2.00E-42 |
| 110 | 7.00E-19 | 1.00E-14 | 3.00E-12 | 4.00E-11 | 3.00E-12 | 8.00E-12 | 6.00E-14 | 4.00E-14 | 2.00E-14 |  | 1.00E-18 | 4.00E-08 |
| 125 | 5.00E-27 | 4.00E-51 | 2.00E-36 | 1.00E-37 | 5.00E-37 | 3.00E-35 |  | 3.00E-44 | 2.00E-36 |  | 7.00E-33 | 6.00E-20 |
| 132 | 3.00E-72 | 1.00E-116 | 6.00E-116 | 6.00E-117 | 3.00E-114 | 2.00E-103 | 5.00E-103 | 8.00E-142 | 5.00E-103 | 9.00E-05 | 3.00E-30 | 1.00E-28 |
| 140 | 1.00E-22 | 3.00E-59 | 1.00E-44 | 3.00E-43 | 2.00E-47 | 8.00E-42 | 3.00E-44 | 5.00E-54 | 5.00E-45 |  | 1.00E-05 | 1.00E-04 |
| 143 | 1.00E-27 | 8.00E-51 | 4.00E-55 | 3.00E-53 | 1.00E-54 | 3.00E-53 |  | 2.00E-68 | 5.00E-53 | 3.00E-53 | 3.00E-12 | 2.00E-07 |
| 180 | 1.00E-19 | 2.00E-85 | 2.00E-132 | 6.00E-130 | 1.00E-121 | 1.00E-127 | 5.00E-136 | 4.00E-162 | 4.00E-136 | 6.00E-128 | 5.00E-05 |  |
| 181 | 3.00E-112 | 0 | 0 | 1.00E-169 | 2.00E-173 | 0 | 0 | 0 | 0 | 0 | 2.00E-09 |  |
| 182 | 7.00E-17 | 1.00E-11 | 7.00E-23 | 3.00E-25 | 4.00E-25 | 3.00E-25 |  | 1.00E-21 | 2.00E-21 |  |  | 2.00E-04 |
| 198 | 3.00E-100 | 2.00E-164 | 7.00E-161 | 3.00E-166 | 3.00E-165 | 6.00E-159 | 4.00E-158 | 1.00E-167 | 1.00E-156 | 5.00E-162 | 7.00E-31 | 5.00E-20 |
| 204 | 1.00E-44 | 2.00E-54 | 1.00E-43 | 9.00E-44 | 2.00E-44 | 5.00E-46 |  | 1.00E-54 | 3.00E-42 |  | 9.00E-34 | 5.00E-14 |
| 215 | 6.00E-80 | 1.00E-154 | 2.00E-164 |  | 4.00E-163 | 1.00E-158 |  | 3.00E-163 | 5.00E-157 |  | 3.00E-35 | 1.00E-38 |
| 288 |  | 3.00E-06 | 0.001 |  |  |  |  |  |  |  | 3.00E-05 |  |
| 327 |  |  |  |  |  | 2.00E-04 |  |  |  | 2.00E-04 |  |  |
| 472 |  |  | 9.00E-06 |  |  |  |  |  |  |  | 1.00E-04 |  |
| 686 | 2.00E-23 | 1.00E-30 | 5.00E-38 | 5.00E-41 | 3.00E-41 | 7.00E-38 |  | 4.00E-40 | 2.00E-32 | 3.00E-38 |  |  |
| 697 | 3.00E-05 | 2.00E-22 | 3.00E-20 | 3.00E-21 | 8.00E-21 | 5.00E-20 |  | 5.00E-19 | 1.00E-22 | 2.00E-20 |  |  |
| 701 | 2.00E-04 |  | 5.00E-04 |  |  |  |  |  |  |  | 3.00E-04 |  |
| Lysis | | | | | | | | | | | | |
| 141 |  |  |  |  |  | 5.00E-04 | 0.001 |  | 0.001 | 4.00E-04 |  |  |
| 699 | 3.00E-21 | 5.00E-65 | 1.00E-39 | 1.00E-39 | 9.00E-41 | 4.00E-40 | 5.00E-40 | 5.00E-44 | 5.00E-40 | 4.00E-40 | 7.00E-19 |  |
| Translation | | | | | | | | | | | | |
| 1 |  |  | 5.00E-30 | 4.00E-24 | 1.00E-25 | 1.00E-23 |  | 2.00E-21 | 1.00E-24 |  |  |  |
| 2 |  |  |  |  |  |  |  |  |  |  |  |  |
| 18 | 2.00E-15 |  | 3.00E-13 | 1.00E-14 | 7.00E-15 | 4.00E-12 |  | 2.00E-15 | 2.00E-13 | 6.00E-12 |  |  |
| 128 |  |  |  |  |  |  |  |  |  |  |  |  |
| 155 |  | 3.00E-04 |  |  |  |  |  |  |  |  |  |  |
| 227 | 5.00E-15 | 6.00E-21 | 3.00E-33 | 9.00E-36 | 1.00E-33 |  |  |  |  |  |  |  |
| 292 |  | 5.00E-170 | 0 | 0 | 0 | 0 | 0 | 0 | 0 |  |  |  |
| 293 |  |  |  |  |  |  |  |  |  |  |  |  |
| 329 |  |  | 5.00E-05 | 3.00E-04 | 1.00E-05 | 3.00E-05 |  |  |  | 1.00E-05 |  |  |
| 492 |  | 6.00E-52 | 6.00E-51 | 1.00E-50 | 2.00E-53 | 1.00E-51 | 7.00E-56 | 8.00E-50 | 3.00E-55 |  |  |  |
| 670 | 3.00E-19 |  | 3.00E-12 | 2.00E-09 | 2.00E-09 | 4.00E-13 |  | 3.00E-08 | 1.00E-12 | 2.00E-13 |  | 1.00E-08 |
| 676 | 5.00E-08 | 5.00E-61 | 1.00E-38 | 1.00E-37 | 2.00E-39 | 3.00E-36 |  | 6.00E-33 | 3.00E-43 |  |  |  |
| 680 | 7.00E-27 | 3.00E-26 | 5.00E-04 |  | 3.00E-04 |  |  |  | 3.00E-07 |  |  |  |
| 705 |  |  |  |  |  |  |  |  |  |  |  |  |
| 708 |  | 9.00E-10 |  | 2.00E-06 | 4.00E-04 |  |  | 6.00E-11 | 3.00E-07 |  |  |  |
| 710 |  | 2.00E-16 |  |  |  |  |  |  |  |  | 2.00E-18 |  |
| Nucleotide Metabolism | | | | | | | | | | | | |
| 121 | 8.00E-10 | 4.00E-09 | 1.00E-05 | 1.00E-05 | 9.00E-06 |  |  | 5.00E-06 |  |  | 3.00E-10 | 8.00E-08 |
| 122 | 5.00E-06 | 3.00E-96 | 1.00E-13 | 4.00E-14 | 2.00E-14 | 4.00E-14 | 7.00E-10 |  | 7.00E-10 | 5.00E-14 | 1.00E-12 | 1.00E-15 |
| 123 |  | 1.00E-89 | 8.00E-09 | 1.00E-10 | 4.00E-13 | 3.00E-09 |  |  | 1.00E-05 | 3.00E-09 |  |  |
| 135 |  |  |  |  |  |  |  |  |  |  | 3.00E-11 |  |
| 195 |  |  | 2.00E-09 | 1.00E-08 | 3.00E-07 | 2.00E-10 | 4.00E-10 | 7.00E-12 | 3.00E-10 | 2.00E-10 |  | 4.00E-46 |
| 221 |  |  | 1.00E-35 | 5.00E-34 | 3.00E-33 | 4.00E-32 |  | 3.00E-43 | 5.00E-33 | 1.00E-32 | 0.001 | 4.00E-46 |
| 238 | 0.001 |  | 7.00E-04 | 4.00E-04 | 3.00E-04 |  |  | 3.00E-04 |  |  |  |  |
| 242 |  |  |  |  |  |  |  |  |  |  |  |  |
| 246 |  |  |  |  |  |  |  |  |  |  | 1.00E-13 |  |
| 252 |  | 3.00E-14 | 0.001 |  |  |  |  |  |  |  |  |  |
| 253 |  |  |  | 0.001 |  |  |  |  |  |  |  |  |
| 261 |  | 7.00E-18 | 9.00E-16 | 7.00E-19 | 2.00E-23 | 8.00E-17 | 5.00E-17 | 1.00E-24 | 1.00E-17 | 5.00E-17 |  | 5.00E-24 |
| 263 |  | 1.00E-74 | 2.00E-61 | 3.00E-69 | 4.00E-67 | 2.00E-65 |  | 5.00E-79 | 1.00E-65 | 2.00E-66 |  |  |
| 266 | 5.00E-06 | 1.00E-08 |  |  |  |  |  | 9.00E-05 |  |  | 4.00E-04 |  |
| 303 | 3.00E-07 | 6.00E-17 | 1.00E-06 | 4.00E-06 | 4.00E-06 |  |  | 1.00E-05 | 2.00E-04 |  |  |  |
| 460 |  | 2.00E-19 |  |  | 7.00E-06 |  |  |  |  |  |  |  |
| 465 |  | 2.00E-25 | 0.001 |  | 2.00E-29 |  |  | 5.00E-09 |  |  |  |  |
| 586 |  |  | 0.001 | 4.00E-05 | 1.00E-06 |  |  | 1.00E-11 |  |  |  |  |
| 587 |  |  | 5.00E-08 | 4.00E-11 | 3.00E-10 |  |  | 4.00E-16 |  |  |  |  |
| 622 |  |  | 3.00E-04 | 2.00E-04 | 3.00E-04 |  |  |  |  |  |  |  |
| 694 | 2.00E-17 | 2.00E-47 | 2.00E-58 | 2.00E-58 | 2.00E-57 | 1.00E-52 |  | 3.00E-46 | 1.00E-49 | 1.00E-52 | 5.00E-12 | 3.00E-06 |
| 709 |  |  | 9.00E-04 | 3.00E-05 | 1.00E-04 |  |  |  |  |  |  | 6.00E-08 |
| **Total** | **84** | **141** | **121** | **117** | **120** | **101** | **31** | **102** | **100** | **63** | **40** | **33** |

**Supplementary Table S3.** T4 core proteins found in Atu_ph07. *Atu_ph07 matches with E-values above 1E-10 are considered “yes” matches and those between 1E-10 and 1E-03 are “unresolved.” Matches with E-values lower than 1E-03 were not considered significant.

| **T4 protein** | **T4 protein function** | **Match in Atu_ph07*** | **Identity (%)** | **E-value** | **Query cover (%)** | **Atu_ph07 protein name** | **Atu_ph07 gp #** |
| --- | --- | --- | --- | --- | --- | --- | --- |
| **Phage morphogenesis** | |  |  |  |  |  |  |
| gp4 | head completion protein | yes | 37 | 6.00E-21 | 92 | head completion protein | 125 |
| gp5 | baseplate lysozyme hub component | unresolved | 26 | 0.001 | 18 | hypothetical protein | 699 |
| gp13 | head completion protein | yes | 20 | 3.00E-08 | 93 | hypothetical protein | 143 |
| gp15 | tail completion protein | yes | 20 | 8.00E-06 | 72 | hypothetical protein | 140 |
| gp17 | subunit of the terminase for DNA packaging | yes; two | 28 | 7.00E-43 | 68 | terminase DNA packaging enzyme large | 109 |
| " |  |  | 31 | 2.00E-08 | 47 | terminase large subunit | 110 |
| gp18 | tail tube subunit | yes; two | 32 | 8.00E-29 | 39 | tail sheath monomer | 132 |
| " |  |  | 37 | 4.00E-05 | 9 | structural protein | 180 |
| gp20 | head portal vertex protein | yes | 25 | 4.00E-21 | 51 | portal vertex of the head | 198 |
| gp21 | prohead core protein and protease | yes | 33 | 8.00E-15 | 66 | prohead core protein | 204 |
| gp22 | prohead core protein | unresolved | 19 | 5.00E-03 | 94 | DNA double-strand break repair Rad50 ATPase | 214 |
| gp23 | precursor of major head protein | yes | 31 | 8.00E-14 | 99 | major capsid protein | 215 |
| gp25 | base plate wedge subunit | yes | 28 | 2.00E-05 | 66 | hypothetical protein | 182 |
| gp34 | proximal tail fiber protein subunit | unresolved | 26 | 4.50E-02 | 7 | EF hand domain/PKD domain protein | 240 |
| gp36 | small distal tail fiber protein subunit | unresolved | 23 | 2.80E-01 | 98 | hypothetical protein | 574 |
| **DNA replication, repair, and recombination** | |  |  |  |  |  |  |
| gp43 | DNA polymerase | yes; two | 26 | 4.00E-27 | 28 | DNA polymerase | 277 |
| " |  |  | 25 | 4.00E-12 | 32 | DNA polymerase | 276 |
| gp44 | sliding clamp loader complex tetramer | yes | 27 | 3.00E-19 | 90 | replication factor C small subunit | 312 |
| gp41 | helicase-primer complex hexamer | yes | 25 | 4.00E-30 | 79 | DNA primase/helicase | 70 |
| gp46 | subunit of a recombination nuclease complex required for initiation of DNA replication | yes; two | 25 | 6.00E-51 | 98 | recombination-related endonuclease | 691 |
| " |  |  | 25 | 1.00E-03 | 29 | hypothetical protein | 108 |
| gp47 | subunit of a recombination nuclease complex required for initiation of DNA replication | yes | 29 | 8.00E-16 | 62 | recombination-related endonuclease | 693 |
| UvsW | recombination DNA-RNA helicase, DNA-dependent ATPase | yes | 23 | 2.00E-27 | 72 | hypothetical protein | 80 |
| **Auxillary metabolism** | |  |  |  |  |  |  |
| nrdA | subunit of an aerobic ribonucleotide reductase complex | yes | 23 | 2.00E-16 | 60 | ribonucleotide reductase of class Ia (aerobic) | 122 |
| nrdB | subunit of an aerobic ribonucleotide reductase complex | unresolved | 21 | 1.00E-03 | 75 | ribonucleotide reductase of class Ia (aerobic) | 123 |
| **Gene expression** | |  |  |  |  |  |  |
| gp55 | sigma factor for late transcription | unresolved | 31 | 2.00E-07 | 50 | hypothetical protein | 694 |

**Supplementary Table S4.** Amino acids, anticodons, and tRNAs encoded in the Atu_ph07 genome.

| **Amino acid** | **Anticodon** | **Number of tRNAs** |
| --- | --- | --- |
| Ala | TGC | 1 |
| Arg | ACG | 1 |
|  | TCT | 1 |
| Asp | GTC | 1 |
| Cys | GCA | 1 |
| Gln | TTG | 2 |
|  | CTG | 1 |
| Glu | TTC | 1 |
| Gly | GCC | 1 |
|  | TCC | 1 |
| His | GTG | 1 |
| Ile | GAT | 1 |
| Leu | TAG | 1 |
|  | CAA | 1 |
| Lys | CCT | 1 |
| Met | CAT | 3 |
| Phe | GAA | 2 |
| Pro | TGG | 3 |
| Ser | TGA | 2 |
|  | GCT | 1 |
| Trp | CCA | 1 |
| Tyr | GTA | 2 |
| Val | TAC | 1 |
| Suppressor | TCA | 1 |

**Supplementary Table S5.** Bacteriophage Atu_ph07 structural proteins identified by ESI-MS/MS

| **Gp #** | **RAST assigned function** | **Updated assigned function** | **Band Nº**  **(most abundant)** | **Protein MW (kDa)** | **Nº of unique peptides** | **Sequence coverage, %** |
| --- | --- | --- | --- | --- | --- | --- |
| *20* | *Putative internalin* |  | *1* | *127,12* | *1* | *1.11* |
| 21 | Hypothetical protein |  | 5 | 39,09 | 3 | 10.50 |
| **31** | **Hypothetical protein** | **Phage tail fiber-like protein** | **1,2,3,4,5,6 (5)** | **45,47** | **9** | **27.70** |
| 32 | Hypothetical protein |  | 1,2,3,4,5,6,7 (7) | 34,14 | 7 | 27.32 |
| 33 | Hypothetical protein |  | 1,2,3,4,5,6,7,8,9,10,11,12,13 (6) | 37,24 | 21 | 64.88 |
| *40* | *Hypothetical protein* |  | *13* | *15,00* | *1* | *8.33* |
| 41 | Hypothetical protein |  | 8,9,13 (13) | 10,62 | 3 | 35.10 |
| **42** | **Conserved phage mega protein** |  | **7,8,9,10,11,12,13 (11)** | **34,60** | **13** | **42.78** |
| 43 | Hypothetical protein |  | 8,9,12,13 (13) | 14,72 | 2 | 24.20 |
| 44 | Hypothetical protein |  | 1,2,3,4,5,6,7,8,9,10,11,12,13 (10) | 86,18 | 21 | 27.33 |
| 46 | Hypothetical protein |  | 13 | 9,14 | 3 | 54.70 |
| 47 | Hypothetical protein |  | 6,7,8,9,10,11,12 (11) | 13,97 | 2 | 22.10 |
| 48 | Hypothetical protein |  | 13 | 9,11 | 3 | 37.00 |
| 50 | Hypothetical protein |  | 1,2,3,4,5,6,7,8,9,10,11,12,13 (11) | 14,36 | 7 | 57.00 |
| 53 | Hypothetical protein |  | 2,3,4,5,6,7,8,9,11,12,13 (9) | 87,08 | 31 | 45.52 |
| 54 | Hypothetical protein |  | 1,2,3,4 (4) | 56,49 | 15 | 30.83 |
| 55 | Hypothetical protein |  | 1,2,5,6,7,8 (8) | 29,65 | 3 | 12.11 |
| 56 | Hypothetical protein |  | 1,3,4,5,6,7,8 (8) | 30,93 | 11 | 46.55 |
| 57 | Hypothetical protein |  | 3,4,5,6,7,8 (8) | 30,16 | 9 | 38.19 |
| 58 | Hypothetical protein |  | 3,4,5,6,7,8 (8) | 29,20 | 7 | 36.52 |
| 59 | Hypothetical protein |  | 3,4,5,6,7 (7) | 31,19 | 7 | 34.10 |
| 60 | Hypothetical protein |  | 4,5,6,7,8,9 (8) | 29,55 | 10 | 42.30 |
| 61 | Hypothetical protein |  | 4,5,7,8 (8) | 29,96 | 9 | 45.30 |
| 62 | Hypothetical protein |  | 1,3,4,5,6,7,8,9 (8) | 29,61 | 12 | 44.40 |
| 63 | Hypothetical protein |  | 1,3,4,5,6,7,8,9,10,11,12,13 (8) | 41,40 | 13 | 38.92 |
| 64 | Hypothetical protein |  | 4,5,6,7,8 (8) | 29,96 | 4 | 17.20 |
| 65 | Hypothetical protein |  | 3,4,5,7 (7) | 32,16 | 10 | 36.80 |
| 67 | Hypothetical protein |  | 1,2,3,4,5,6,7,8,9,10,11,12,13 (8) | 26,32 | 16 | 66.98 |
| 68 | Hypothetical protein |  | 9,11,12,13 (13) | 9,95 | 5 | 63.82 |
| 69 | Hypothetical protein |  | 13 | 11,73 | 1 | 11.80 |
| *72* | *Hypothetical protein* |  | *8* | *27,59* | *1* | *4.60* |
| 76 | Hypothetical protein |  | 11,12 (12) | 14,88 | 4 | 33.90 |
| *106* | *Hypothetical protein* | RecA-like protein | *3,5 (5)* | *40,53* | *1* | *3.26* |
| 107 | Hypothetical protein | ssDNA-binding protein | 5,6,7,8,11,13 (7) | 38,06 | 3 | 13.21 |
| 108 | Hypothetical protein |  | 1,2,3,4,5,6,7,8,9,10,11,12,13 (13) | 97,96 | 25 | 31.24 |
| **109** | **Gp17 terminase DNA packaging enzyme large subunit** |  | **11,12 (11)** | **68,13** | **4** | **10.46** |
| ***110*** | ***Phage terminase large subunit*** |  | ***12,13 (13)*** | ***14,29*** | ***1*** | ***7.32*** |
| 111 | Hypothetical protein |  | 9,13 (13) | 12,80 | 2 | 23.90 |
| 112 | Autotransporter |  | 1,2,3,4,5 (2) | 76,62 | 15 | 29.56 |
| 113 | Hypothetical protein |  | 1,2,3,4,5,6,7,8,9,11,12,13 (7) | 25,06 | 11 | 61.70 |
| *122* | *Ribonucleotide reductase of class Ia (aerobic) alpha subunit (EC 1.17.4.1)* |  | *3* | *62,00* | *1* | *1.83* |
| ***125*** | ***Phage head completion protein*** |  | ***13*** | ***18,50*** | ***1*** | ***8.12*** |
| 126 | Hypothetical protein |  | 4,6 (6) | 36,51 | 4 | 15.20 |
| 127 | Hypothetical protein |  | 1,2,3,4,5,6,7,8,9, 12,13 (9) | 21,37 | 7 | 52.83 |
| **132** | **Phage tail sheath monomer** |  | **1,2,3,4,5,6,7,8,9,10,11,12,13 (2)** | **117,53** | **31** | **41.14** |
| *133* | *Hypothetical protein* |  | *9* | *20,79* | *1* | *4.86* |
| **140** | **Hypothetical protein** | **Tail sheath stabilizer and completion protein** | **1,2,3,4,5,8 (4)** | **51,67** | **9** | **26.91** |
| 142 | Hypothetical protein |  | 3,4,5,6 (5) | 41,07 | 3 | 10.20 |
| **143** | **Hypothetical protein** | **Head completion adaptor; neck** | **3,4,5,6,7 (7)** | **28,45** | **4** | **18.40** |
| 170 | Hypothetical protein |  | 1,2,3,4,5,6,7,8,9 (1) | 31,89 | 8 | 35.16 |
| 176 | Hypothetical protein |  | 1,2,3,4,5,6,7 (4) | 66,07 | 14 | 36.20 |
| 177 | Hypothetical protein |  | 1,2,3,4,6,7,8,9,10,11 (11) | 21,66 | 2 | 11.60 |
| **181** | **Hypothetical protein** | **Baseplate wedge** | **1,2,3 (2)** | **136,79** | **11** | **11.85** |
| **182** | **Hypothetical protein** | **Baseplate wedge** | **12,13 (12)** | **15,07** | **3** | **22.50** |
| *183* | *Hypothetical protein* |  | *9* | *21,97* | *1* | *4.74* |
| 185 | Hypothetical protein |  | 13 | 10,13 | 2 | 12.80 |
| 188 | Hypothetical protein |  | 7,8 (8) | 18,34 | 4 | 26.20 |
| 196 | Hypothetical protein |  | 1,2,3,4,13 (2) | 57,94 | 14 | 30.20 |
| 197 | Hypothetical protein |  | 1,2,3,4,5 (1) | 87,30 | 15 | 23.12 |
| **198** | **Phage portal vertex of the head T4-like phage Gp20** |  | **1,2,3,4,5 (3)** | **66,31** | **20** | **43.00** |
| 199 | Hypothetical protein |  | 12,13 | 9,01 | 1 | 27.30 |
| 200 | Topoisomerase IV subunit A (EC 5.99.1-) |  | 3,4,5,6,7,8,9,10,11,12,13 (9) | 44,81 | 4 | 16.96 |
| **204** | **Phage prohead core protein** |  | **1,2,3,4,5,6,7,8,9,10,11,12,13 (11)** | **22,97** | **7** | **36.20** |
| 205 | Hypothetical protein |  | 4,5,6,7,8,9,10,11,12,13 (9) | 29,01 | 10 | 43.60 |
| 206 | Hypothetical protein |  | 4,5,6,7,8,9 (8) | 25,47 | 6 | 28.00 |
| 210 | Hypothetical protein |  | 13 | 8,75 | 1 | 20.30 |
| 213 | MJ0042 family fingerlike protein |  | 2,3,4,5,7,8,9,10,11,12,13 (13) | 32,88 | 7 | 20.60 |
| **215** | **Phage major capsid protein of Caudovirales (T4-like gp23)** |  | **1,2,3,4,5,6,7,8,9,10,11,12,13 (6)** | **43,51** | **10** | **40.56** |
| *238* | *ADP-ribose pyrophosphatase (EC_3_6_1_13)* |  | *9* | *18,68* | *1* | *6.06* |
| 239 | Hypothetical protein |  | 1,4,5,6,7,8,9,10,11,12 (11) | 15,80 | 3 | 40.60 |
| 240 | EF hand domain PKD domain protein |  | 5,6 (5) | 36,31 | 2 | 13.90 |
| 241 | Hypothetical protein |  | 1,2,3,4,5,6 (6) | 43,70 | 6 | 18.24 |
| 250 | Hypothetical protein |  | 7,8,13 (13) | 26,25 | 2 | 7.66 |
| 257 | Hypothetical protein |  | 1,2,3,5,6,7,8,9,10,11,12 (11) | 16,73 | 4 | 43.26 |
| *264* | *Hypothetical protein* |  | *5,7 (5)* | *46,80* | *2* | *4,56* |
| *275* | *Hypothetical protein* |  | *2,5,7,8 (8)* | *27,85* | *1* | *3.63* |
| 284 | Hypothetical protein |  | 1,3,4,5,6,7,8,9 (8) | 25,12 | 7 | 50.20 |
| 285 | Hypothetical protein |  | 12 | 14,30 | 3 | 23.60 |
| 286 | Hypothetical protein |  | 2,3,4,5 (4) | 38,73 | 8 | 33.29 |
| 287 | Hypothetical protein |  | 1,2,3,4,5,6,7,8 (5) | 38,49 | 4 | 18.70 |
| **288** | **Phage tail fiber protein** |  | **1,2,3,4,5,6,7,8,9,10,11,12,13 (2)** | **61,03** | **14** | **35.90** |
| 289 | Hypothetical protein |  | 5,7,8,9,11,12,13 (13) | 12,51 | 2 | 26.40 |
| *290* | *Hypothetical protein* |  | *12* | *15,56* | *1* | *15.20* |
| 294 | Hypothetical protein |  | 4,5,7,8,9,10,11,12,13 (13) | 12,38 | 4 | 42.20 |
| 295 | Hypothetical protein |  | 11 | 14,92 | 3 | 18.90 |
| 303 | Probable NUDIX hydrolase |  | 3,11 (11) | 14,75 | 1 | 10.00 |
| 305 | Hypothetical protein |  | 12 | 27,33 | 2 | 8.75 |
| 306 | Hypothetical protein |  | 9,10,12 (9) | 20,39 | 2 | 16.40 |
| 311 | Hypothetical protein |  | 13 | 11,83 | 3 | 42.30 |
| 314 | Hypothetical protein |  | 12 | 12,81 | 1 | 16.20 |
| 320 | Streptococcal hemagglutinin protein |  | 1,3,4,5,6 (6) | 40,73 | 4 | 18.80 |
| 321 | Hypothetical protein |  | 1,2,3,4 (1) | 36,02 | 2 | 8.81 |
| 322 | Hypothetical protein |  | 2,4,6,7 (6,7) | 33,63 | 2 | 7.3 |
| 323 | Hypothetical protein |  | 1,2,3,4,5,6,7,8,9,11,13 (6) | 42,40 | 17 | 62.20 |
| 324 | Hypothetical protein |  | 1,2,3,4,5,6,7 (7) | 34,76 | 6 | 26.50 |
| 325 | Hypothetical protein |  | 1,2,3,4 (3) | 77,23 | 13 | 26.03 |
| 326 | Hypothetical protein |  | 1,2,3,4,5 (1) | 75,68 | 9 | 16.70 |
| **327** | **Phage tail fiber protein** |  | **1,2,3,4,5,6,7,8,9 (2)** | **72,15** | **14** | **37.40** |
| 328 | Hypothetical protein |  | 1,2,7,9 (1) | 93,95 | 8 | 12.74 |
| *329* | *Extracellular serine proteinase precursor (EC 3.4.21-)* |  | *1,2,13 (1)* | *139,38* | *3* | *2.08* |
| 377 | Hypothetical protein |  | 1,2,3,4,5 (4) | 49,73 | 6 | 16.70 |
| 381 | Hypothetical protein |  | 11 | 20,00 | 2 | 15.90 |
| *390* | *Hypothetical protein* |  | *13* | *11,75* | *1* | *9.00* |
| 393 | Phage protein |  | 12,13 (13) | 11,30 | 2 | 29.10 |
| 394 | No significant homology |  | 1,2,3,4,8 (4) | 55,10 | 18 | 41.01 |
| 407 | Hypothetical protein |  | 1,2,3,4,5,6,7,8 (6) | 39,67 | 17 | 44.40 |
| 408 | Hypothetical protein |  | 1,2,3,4,5,6,7,8,9 (8) | 30,60 | 6 | 24.78 |
| *443* | *Hypothetical protein* |  | *8,12 (12)* | *13,04* | *1* | *7.48* |
| 466 | Hypothetical protein |  | 1,2,3,4,5,6,7,8,9,11,12,13 (4) | 52,32 | 21 | 62.80 |
| 470 | Hypothetical protein |  | 13 | 14,60 | 2 | 18.50 |
| **472** | **Phage tail fiber protein** |  | **1,2,3 (2)** | **80,58** | **14** | **28.10** |
| 475 | Hypothetical protein |  | 1,2,3,4,5,6,7,8,9,10,11,12,13 (11) | 31,66 | 14 | 39.54 |
| 482 | Hypothetical protein |  | 7,8,9,10,11,12,13 (12) | 31,68 | 9 | 36.70 |
| 483 | Hypothetical protein |  | 5,6,7,8,9,10,11,12 (12) | 33,72 | 16 | 55.80 |
| 487 | Hypothetical protein |  | 1,4,5,6,7,8,9,10,11,12,13 (13) | 12,99 | 8 | 50.90 |
| 498 | Hypothetical protein |  | 1,2,3,4,5,6,7,8,9,10,11,12,13 (7) | 35,16 | 23 | 75.50 |
| 514 | Hypothetical protein |  | 12,13 (13) | 7,10 | 2 | 31.70 |
| 544 | Hypothetical protein |  | 4,5,6,7,8,9,10,12,13 (13) | 31,80 | 9 | 32.06 |
| 548 | Hypothetical protein |  | 12 | 13,65 | 3 | 40.00 |
| 561 | Hypothetical protein |  | 1,2,3,4,5,6,7,8,9,10,11,13 (9) | 17,79 | 7 | 52.16 |
| 574 | Hypothetical protein |  | 1,5 (5) | 52,57 | 6 | 18.10 |
| 576 | Hypothetical protein |  | 1,4,5 (4) | 52,20 | 4 | 7.23 |
| 577 | Hypothetical protein |  | 1,2,3,4,5,6,7,8,9,10,11,12,13 (12) | 14,42 | 5 | 52.10 |
| 578 | Hypothetical protein |  | 2,3,4,5,6,7,8,9,10,11 (9) | 19,16 | 4 | 43.90 |
| 594 | Hypothetical protein |  | 3,7,8,9,12,13 (13) | 23,87 | 15 | 57.51 |
| *606* | *Hypothetical protein* |  | *13* | *8,62* | *1* | *13.90* |
| *616* | *Hypothetical protein* |  | *1* | *32,06* | *1* | *4.67* |
| 624 | Putative phage related protein precursor | D-alanyl-D-alanine carboxypeptidase | 12,13 (12) | 14,79 | 1 | 9.85 |
| *627* | *Hypothetical protein* |  | *8* | *27,46* | *1* | *3.69* |
| 636 | Hypothetical protein |  | 13 | 11,55 | 2 | 21.60 |
| 639 | Hypothetical protein |  | 12 | 16,38 | 3 | 29.60 |
| 661 | Hypothetical protein |  | 12 | 14,72 | 1 | 10.40 |
| 668 | Hypothetical protein |  | 1,2,3,4,5,6,7,8,9,10,11,12 (11) | 20,34 | 8 | 50.59 |
| 672 | Hypothetical protein |  | 9,10 (9) | 22,56 | 3 | 23.15 |
| 675 | Hypothetical protein |  | 1,2,3,4,5,6,7,8,9,10,11,12,13 (8) | 24,87 | 3 | 20.60 |
| 696 | Hypothetical protein |  | 2,3,4,5,7,8 (4) | 50,11 | 5 | 13.90 |
| **697** | **Hypothetical protein** | **Baseplate wedge** | **9,10,11,12,13 (13)** | **12,32** | **3** | **38.50** |
| 698 | Hypothetical protein |  | 1,2,3 (3) | 72,75 | 7 | 11.50 |
| **701** | **PE-PGRS virulence associated protein** | **Tail tape measure protein** | **5,6,7,8,9 (9)** | **21,77** | **8** | **68.80** |
| *707* | *Hypothetical protein* |  | *9* | *24,28* | *1* | *3.98* |

*^a^Italics:* Proteins with only a single peptide or low coverage **Bold: Proteins annotated as structural proteins within the head, neck, tail or tail fibers**
